# Supplementary material for: A systematic review of methodological approaches for evaluating real-world effectiveness of COVID-19 vaccines: Advising resource-constrained settings
Source: PLoS One. 2022 Jan 11;17(1):e0261930. doi: 10.1371/journal.pone.0261930 (PMC8752025; doi:10.1371/journal.pone.0261930)
Supplement: S1 Appendix — (DOCX) [file pone.0261930.s001.docx]

**Search terms in Medline**

"COVID-19 Vaccines"[MeSH Terms] AND ("effect"[All Fields] OR "effecting"[All Fields] OR "effective"[All Fields] OR "effectively"[All Fields] OR "effectiveness"[All Fields] OR "effectivenesses"[All Fields] OR "effectives"[All Fields] OR "effectivities"[All Fields] OR "effectivity"[All Fields] OR "effects"[All Fields] OR "nationwide"[All Fields] OR "real world"[All Fields] OR "post approval"[All Fields] OR "post marketing"[All Fields] OR ("cohort studies"[MeSH Terms] OR ("cohort"[All Fields] AND "studies"[All Fields]) OR "cohort studies"[All Fields] OR "cohort"[All Fields] OR "cohort s"[All Fields] OR "cohorte"[All Fields] OR "cohorts"[All Fields]) OR "adverse event"[All Fields] OR "side effect"[All Fields])

**Search terms in Scopus**

( ( TITLE-ABS-KEY ( covid-19 ) ) OR ( TITLE-ABS-KEY ( sars-cov-2 ) ) OR ( TITLE-ABS-KEY ( "coronavirus Disease 2019" ) ) OR ( TITLE-ABS-KEY ( "Severe acute respiratory syndrome coronavirus 2 " ) ) ) AND ( ( TITLE-ABS-KEY ( vaccine ) ) OR ( TITLE-ABS-KEY ( vaccination ) ) OR ( TITLE-ABS-KEY ( immnunization ) ) ) AND ( ( ( TITLE-ABS-KEY ( effectiveness ) ) OR ( TITLE-ABS-KEY ( nationwide ) ) OR ( TITLE-ABS-KEY ( "real world" ) ) ) OR ( TITLE-ABS-KEY ( "post approval" ) ) OR ( TITLE-ABS-KEY ( "post marketing" ) ) OR ( TITLE-ABS-KEY ( cohort ) ) OR ( TITLE-ABS-KEY ( "adverse event" ) ) OR ( TITLE-ABS-KEY ( "side effect" ) ) )

**Studies excluded at full text screening**

Abu-Hammad O, Alduraidi H, Abu-Hammad S, Alnazzawi A, Babkair H, Abu-Hammad A et al. Side Effects Reported by Jordanian Healthcare Workers Who Received COVID-19 Vaccines. Vaccines. 2021;9(6):577. *Excluded based on outcome (adverse events).*

Abu Jabal K, Ben-Amram H, Beiruti K, Batheesh Y, Sussan C, Zarka S et al. Impact of age, ethnicity, sex and prior infection status on immunogenicity following a single dose of the BNT162b2 mRNA COVID-19 vaccine: real-world evidence from healthcare workers, Israel, December 2020 to January 2021. Eurosurveillance. 2021;26(6). *Excluded based on outcome (immunogenicity).*

Blumenthal K, Robinson L, Camargo C, Shenoy E, Banerji A, Landman A et al. Acute Allergic Reactions to mRNA COVID-19 Vaccines. JAMA. 2021;325(15):1562. *Excluded based on outcome (adverse events).*

Boyarsky B, Werbel W, Avery R, Tobian A, Massie A, Segev D et al. Immunogenicity of a Single Dose of SARS-CoV-2 Messenger RNA Vaccine in Solid Organ Transplant Recipients. JAMA. 2021;325(17):1784. *Excluded based on outcome (immunogenicity).*

Callegaro A, Borleri D, Farina C, Napolitano G, Valenti D, Rizzi M et al. Antibody response to SARS‐CoV‐2 vaccination is extremely vivacious in subjects with previous SARS‐CoV‐2 infection. Journal of Medical Virology. 2021;93(7):4612-4615. *Excluded based on outcome (immunogenicity).*

Chen G, Li X, Sun M, Zhou Y, Yin M, Zhao B et al. COVID-19 mRNA Vaccines Are Generally Safe in the Short Term: A Vaccine Vigilance Real-World Study Says. Frontiers in Immunology. 2021;12. *Excluded based on outcome (adverse events).*

Christie A, Henley S, Mattocks L, Fernando R, Lansky A, Ahmad F et al. Decreases in COVID-19 Cases, Emergency Department Visits, Hospital Admissions, and Deaths Among Older Adults Following the Introduction of COVID-19 Vaccine — United States, September 6, 2020–May 1, 2021. MMWR Morbidity and Mortality Weekly Report. 2021;70(23):858-864. *Excluded based on outcome (not effectiveness).*

Cirillo N. Reported orofacial adverse effects of COVID‐19 vaccines: The knowns and the unknowns. Journal of Oral Pathology & Medicine. 2021;50(4):424-427. *Excluded based on outcome (adverse events).*

Collier A, McMahan K, Yu J, Tostanoski L, Aguayo R, Ansel J et al. Immunogenicity of COVID-19 mRNA Vaccines in Pregnant and Lactating Women. JAMA. 2021;325(23):2370. *Excluded based on outcome (immunogenicity).*

Damiani G, Allocco F, Malagoli P. COVID‐19 vaccination and patients with psoriasis under biologics: real‐life evidence on safety and effectiveness from Italian vaccinated healthcare workers. Clinical and Experimental Dermatology. 2021;46(6):1106-1108. *Excluded based on outcome (not effectiveness).*

Dean N. Hospital admissions due to COVID-19 in Scotland after one dose of vaccine. The Lancet. 2021;397(10285):1601-1603. *Excluded based on outcome (not effectiveness).*

Fertel B, Milk J, Simon E, Muir M, Smalley C. COVID-19 vaccine adverse reactions bring patients to emergency departments. The American Journal of Emergency Medicine. 2021;. *Excluded based on outcome (adverse events).*

Frenck R, Klein N, Kitchin N, Gurtman A, Absalon J, Lockhart S et al. Safety and Efficacy of the BNT162b2 mRNA Covid-19 Vaccine. New England Journal of Medicine. 2021;384(16):1576-1578. *Excluded as wrong study type (not effectiveness study).*

Friedrich S, Friede T. Causal inference methods for small non-randomized studies: Methods and an application in COVID-19. Contemporary Clinical Trials. 2020;99:106213. *Excluded based on article type (not primary research).*

Gee J, Marquez P, Su J, Calvert G, Liu R, Myers T et al. First Month of COVID-19 Vaccine Safety Monitoring — United States, December 14, 2020–January 13, 2021. MMWR Morbidity and Mortality Weekly Report. 2021;70(8):283-288. *Excluded based on outcome (not effectiveness).*

Geers D, Shamier M, Bogers S, den Hartog G, Gommers L, Nieuwkoop N et al. SARS-CoV-2 variants of concern partially escape humoral but not T-cell responses in COVID-19 convalescent donors and vaccinees. Science Immunology. 2021;6(59):eabj1750. *Excluded based on outcome (immunogenicity).*

Hodgson S, Mansatta K, Mallett G, Harris V, Emary K, Pollard A. What defines an efficacious COVID-19 vaccine? A review of the challenges assessing the clinical efficacy of vaccines against SARS-CoV-2. The Lancet Infectious Diseases. 2021;21(2):e26-e35. *Excluded based on article type (not primary research).*

Lee E, Cines D, Gernsheimer T, Kessler C, Michel M, Tarantino M et al. Thrombocytopenia following Pfizer and Moderna SARS‐CoV ‐2 vaccination. American Journal of Hematology. 2021;96(5):534-537. *Excluded based on outcome (adverse events).*

Lee Y, Lim S, Lee J, Lim J, Kim M, Kwon S et al. Adverse Reactions of the Second Dose of the BNT162b2 mRNA COVID-19 Vaccine in Healthcare Workers in Korea. Journal of Korean Medical Science. 2021;36(21). *Excluded based on outcome (adverse events).*

Levine-Tiefenbrun M, Yelin I, Katz R, Herzel E, Golan Z, Schreiber L et al. Initial report of decreased SARS-CoV-2 viral load after inoculation with the BNT162b2 vaccine. Nature Medicine. 2021;27(5):790-792. *Excluded based on outcome (viral load).*

Lewnard J, Patel M, Jewell N, Verani J, Kobayashi M, Tenforde M et al. Theoretical Framework for Retrospective Studies of the Effectiveness of SARS-CoV-2 Vaccines. Epidemiology. 2021;32(4):508-517. *Excluded based on article type (not primary research).*

Lin T, Liao S, Lai C, Paci E, Chuang S. Effectiveness of non-pharmaceutical interventions and vaccine for containing the spread of COVID-19: Three illustrations before and after vaccination periods. Journal of the Formosan Medical Association. 2021;120:S46-S56. *Excluded based on article type (modelling study).*

Lu J, Wen X, Guo Q, Ji M, Zhang F, Wagner A et al. Sensitivity to COVID-19 Vaccine Effectiveness and Safety in Shanghai, China. Vaccines. 2021;9(5):472. *Excluded based on outcome (attitudes).*

Mateo-Urdiales A, Del Manso M, Andrianou X, Spuri M, D'Ancona F, Filia A et al. Initial impact of SARS-Cov-2 vaccination on healthcare workers in Italy– Update on the 28th of March 2021. Vaccine. 2021;39(34):4788-4792. *Excluded based on outcome (not effectiveness).*

Milman O, Yelin I, Aharony N, Katz R, Herzel E, Ben-Tov A et al. Community-level evidence for SARS-CoV-2 vaccine protection of unvaccinated individuals. Nature Medicine. 2021;. *Excluded based on outcome (not effectiveness).*

Monin L, Laing A, Muñoz-Ruiz M, McKenzie D, del Molino del Barrio I, Alaguthurai T et al. Safety and immunogenicity of one versus two doses of the COVID-19 vaccine BNT162b2 for patients with cancer: interim analysis of a prospective observational study. The Lancet Oncology. 2021;22(6):765-778. *Excluded based on outcome (adverse events and immunogenicity).*

Patel M, Bergeri I, Bresee J, Cowling B, Crowcroft N, Fahmy K et al. Evaluation of post-introduction COVID-19 vaccine effectiveness: Summary of interim guidance of the World Health Organization. Vaccine. 2021;39(30):4013-4024. *Excluded based on article type (not primary research).*

Salmerón Ríos S, Mas Romero M, Cortés Zamora E, Tabernero Sahuquillo M, Romero Rizos L, Sánchez‐Jurado P et al. Immunogenicity of the BNT162b2 vaccine in frail or disabled nursing home residents: COVID‐A study. Journal of the American Geriatrics Society. 2021;69(6):1441-1447. *Excluded based on outcome (immunogenicity).*

Sheikh A, McMenamin J, Taylor B, Robertson C. SARS-CoV-2 Delta VOC in Scotland: demographics, risk of hospital admission, and vaccine effectiveness. The Lancet. 2021;. *Sub-group analysis of another included study.*

Song J, Cheong H, Kim S, Lee S, Kim S, Noh J et al. Early Safety Monitoring of COVID-19 Vaccines in Healthcare Workers. Journal of Korean Medical Science. 2021;36(15). *Excluded based on outcome (adverse events).*

Sørvoll I, Horvei K, Ernstsen S, Lægreid I, Lund S, Grønli R et al. An observational study to identify the prevalence of thrombocytopenia and anti‐PF4/polyanion antibodies in Norwegian health care workers after COVID‐19 vaccination. Journal of Thrombosis and Haemostasis. 2021;19(7):1813-1818. *Excluded based on outcome (adverse events).*

Tanislav C, El Ansari T, Meyer M, Müller M, Grübener R, Stein T et al. Effect of SARS-CoV-2 vaccination among health care workers in a geriatric care unit after a B.1.1.7-variant outbreak. Public Health. 2021;198:e20-e22.

Tougeron D, Hentzien M, Seitz-Polski B, Bani-Sadr F, Bourhis J, Ducreux M et al. Severe acute respiratory syndrome coronavirus 2 vaccination for patients with solid cancer: Review and point of view of a French oncology intergroup (GCO, TNCD, UNICANCER). European Journal of Cancer. 2021;150:232-239. *Excluded based on article type (not primary research).*

Victor P, Mathews K, Paul H, Mammen J, Murugesan M. Protective Effect of COVID-19 Vaccine Among Health Care Workers During the Second Wave of the Pandemic in India. Mayo Clinic Proceedings. 2021;96(9):2493-2494. *Excluded as insufficient details on methodology.*

Vilches T, Zhang K, Van Exan R, Langley J, Moghadas S. Projecting the impact of a two-dose COVID-19 vaccination campaign in Ontario, Canada. Vaccine. 2021;39(17):2360-2365. *Excluded based on article type (modelling study).*

illiams S, Vusirikala A, Ladhani S, Fernandez Ruiz De Olano E, Iyanger N, Aiano F et al. An outbreak caused by the SARS-CoV-2 Delta (B.1.617.2) variant in a care home after partial vaccination with a single dose of the COVID-19 vaccine Vaxzevria, London, England, April 2021. Eurosurveillance. 2021;26(27). *Excluded based on outcomes (not vaccine effectiveness).*

Zhao H, Souders C, Carmel M, Anger J. Low Rates of Urologic Side Effects Following Coronavirus Disease Vaccination: An Analysis of the Food and Drug Administration Vaccine Adverse Event Reporting System. Urology. 2021;153:11-13. *Excluded based on outcome (adverse events).*
