# Supplementary material for: A systematic review of methodological approaches for evaluating real-world effectiveness of COVID-19 vaccines: Advising resource-constrained settings
Source: PLoS One. 2022 Jan 11;17(1):e0261930. doi: 10.1371/journal.pone.0261930 (PMC8752025; doi:10.1371/journal.pone.0261930)
Supplement: Attachment — Submitted filename: PONE-D-21-26919_reviewer.pdf [file pone.0261930.s004.pdf]

PLOS ONE

A systematic review of methodological approaches for evaluating real-world effectiveness of COVID-19 vaccines: advising resource-constrained settings

--Manuscript Draft--

Manuscript Number:	PONE-D-21-26919
Article Type:	Research Article
Full Title:	A systematic review of methodological approaches for evaluating real-world effectiveness of COVID-19 vaccines: advising resource-constrained settings
Short Title:	Systematic review of real-world COVID-19 vaccine effectiveness study methods and guidance for LMICs
Corresponding Author:	Siobhan Botwright<br>Health Intervention and Technology Assessment Program<br>THAILAND
Keywords:	Vaccination; COVID-19; real-world effectiveness; observational studies; vaccine effectiveness
Abstract:	Background<br>Real-world effectiveness studies are important for monitoring performance of COVID-19 vaccination programmes and informing COVID-19 prevention and control policies. We aimed to synthesise methodological approaches used in COVID-19 vaccine effectiveness studies, in order to evaluate which approaches are most appropriate to implement in low- and middle-income countries (LMICs).<br>Methods<br>For this rapid systematic review, we searched PubMed and Scopus for articles published from inception to July 7, 2021, without language restrictions. We included any type of peer-reviewed observational study measuring COVID19 vaccine effectiveness, for any population. We excluded randomised control trials and modelling studies. All data used in the analysis were extracted from included papers. We used a standardised data extraction form, modified from STrengthening the Reporting of OBservational studies in Epidemiology (STROBE). Study quality was assessed using the REal Life EVidence AssessmeNt Tool (RELEVANT) tool. This study is registered with PROSPERO, CRD42021264658.<br>Results<br>Our search identified 3,214 studies, of which 26 were eligible for analysis. All studies were conducted in 7 highincome countries and the majority assessed mRNA vaccines (81% mRNA only, 15% mRNA and viral vector). Twenty-one of the 26 studies (81%) used a cohort study design. There was significant heterogeneity for full vaccination effectiveness estimates across studies (infection: n=17, mean=79%; hospitalisation: n=7, mean=89%; death: n=3, mean=92%). Follow-up time for all studies was short (mean=9.5 weeks). Across studies, short follow-up time and limited assessment and mitigation of potential confounders, including previous SARS-CoV-2 infection and healthcare seeking behaviour, were major limitations.<br>Discussion<br>This review summarises methodological approaches for evaluating real-world effectiveness of COVID-19 vaccines and highlights the lack of such studies in LMICs, as well as the importance of context-specific vaccine effectiveness data. Further research in LMICs will refine guidance for conducting real-world COVID-19 vaccine effectiveness studies in resource-constrained settings.
Order of Authors:	Yot Teerawattananon, PhD
	Thunyarat Anothaisintawee
	Chatkamol Pheerapanyawaranun
	Siobhan Botwright
	Katika Akksilp
	Natchalaikorn Sirichumroonwit

Powered by Editorial Manager® and ProduXion Manager® from Aries Systems Corporation

	Wanrudee Isaranuwatchai
Additional Information:	
**Question**	**Response**
**Financial Disclosure**<br><br>Enter a financial disclosure statement that describes the sources of funding for the work included in this submission. Review the submission guidelines for detailed requirements. View published research articles from *PLOS ONE* for specific examples.<br><br>This statement is required for submission and **will appear in the published article** if the submission is accepted. Please make sure it is accurate.<br><br>**Unfunded studies**<br>Enter: *The author(s) received no specific funding for this work.*<br><br>**Funded studies**<br>Enter a statement with the following details:<br>• Initials of the authors who received each award<br>• Grant numbers awarded to each author<br>• The full name of each funder<br>• URL of each funder website<br>• Did the sponsors or funders play any role in the study design, data collection and analysis, decision to publish, or preparation of the manuscript?<br>• **NO** - Include this sentence at the end of your statement: *The funders had no role in study design, data collection and analysis, decision to publish, or preparation of the manuscript.*<br>• **YES** - Specify the role(s) played.<br><br>* typeset	This study was funded by the Health Systems Research Institute (https://hsri.or.th/researcher), grant number 64134002RM011L0.<br><br>The funder of the study had no role in study design, data collection, data analysis, data interpretation, or writing of the report.
**Competing Interests**<br><br>Use the instructions below to enter a competing interest statement for this submission. On behalf of all authors, disclose any competing interests that could be perceived to bias this work—acknowledging all financial support and any other relevant financial or non-financial competing interests.	This study was funded by the Health Systems Research Institute (https://hsri.or.th/researcher), grant number 64134002RM011L0. The authors declare that no other competing interests exist.

This statement is required for submission and **will appear in the published article** if the submission is accepted. Please make sure it is accurate and that any funding sources listed in your Funding Information later in the submission form are also declared in your Financial Disclosure statement.<br><br>View published research articles from *PLOS ONE* for specific examples.<br><br>**NO authors have competing interests**<br><br>Enter: *The authors have declared that no competing interests exist.*<br><br>**Authors with competing interests**<br><br>Enter competing interest details beginning with this statement:<br><br>*I have read the journal's policy and the authors of this manuscript have the following competing interests: [insert competing interests here]*<br><br>* typeset	
**Ethics Statement**<br><br>Enter an ethics statement for this submission. This statement is required if the study involved:<br><br>• Human participants<br>• Human specimens or tissue<br>• Vertebrate animals or cephalopods<br>• Vertebrate embryos or tissues<br>• Field research<br><br>Write "N/A" if the submission does not require an ethics statement.<br><br>General guidance is provided below. Consult the submission guidelines for detailed instructions. **Make sure that all**	N/A

**information entered here is included in the Methods section of the manuscript.**<br><br>**Format for specific study types**<br><br>**Human Subject Research (involving human participants and/or tissue)**<br>• Give the name of the institutional review board or ethics committee that approved the study<br>• Include the approval number and/or a statement indicating approval of this research<br>• Indicate the form of consent obtained (written/oral) or the reason that consent was not obtained (e.g. the data were analyzed anonymously)<br><br>**Animal Research (involving vertebrate animals, embryos or tissues)**<br>• Provide the name of the Institutional Animal Care and Use Committee (IACUC) or other relevant ethics board that reviewed the study protocol, and indicate whether they approved this research or granted a formal waiver of ethical approval<br>• Include an approval number if one was obtained<br>• If the study involved *non-human primates*, add *additional details* about animal welfare and steps taken to ameliorate suffering<br>• If anesthesia, euthanasia, or any kind of animal sacrifice is part of the study, include briefly which substances and/or methods were applied<br><br>**Field Research**<br><br>Include the following details if this study involves the collection of plant, animal, or other materials from a natural setting:<br>• Field permit number<br>• Name of the institution or relevant body that granted permission	
**Data Availability**<br><br>Authors are required to make all data underlying the findings described fully available, without restriction, and from the time of publication. PLOS allows rare exceptions to address legal and ethical	Yes - all data are fully available without restriction

concerns. See the PLOS Data Policy and FAQ for detailed information.<br><br>A Data Availability Statement describing where the data can be found is required at submission. Your answers to this question constitute the Data Availability Statement and **will be published in the article**, if accepted.<br><br>**Important:** Stating 'data available on request from the author' is not sufficient. If your data are only available upon request, select 'No' for the first question and explain your exceptional situation in the text box.<br><br>Do the authors confirm that all data underlying the findings described in their manuscript are fully available without restriction?	
**Describe where the data may be found in full sentences. If you are copying our sample text, replace any instances of XXX with the appropriate details.**<br><br>• If the data are **held or will be held in a public repository**, include URLs, accession numbers or DOIs. If this information will only be available after acceptance, indicate this by ticking the box below. For example: *All XXX files are available from the XXX database (accession number(s) XXX, XXX.*).<br>• If the data are all contained **within the manuscript and/or Supporting Information files**, enter the following: *All relevant data are within the manuscript and its Supporting Information files.*<br>• If neither of these applies but you are able to provide **details of access elsewhere**, with or without limitations, please do so. For example:<br><br>*Data cannot be shared publicly because of [XXX]. Data are available from the XXX Institutional Data Access / Ethics Committee (contact via XXX) for researchers who meet the criteria for access to confidential data.*	All articles included in the review are listed in the manuscript and excluded articles are available in supplementary file. Template data extraction form and data extraction for all included studies will be made available on request.

The data underlying the results presented in the study are available from (include the name of the third party and contact information or URL).<br>• This text is appropriate if the data are owned by a third party and authors do not have permission to share the data.<br><br>* typeset	
Additional data availability information:	

Powered by Editorial Manager® and ProduXion Manager® from Aries Systems Corporation

Manuscript

Click here to access/download;Manuscript;RWE c19 vaccines - FULL - 16Aug2021.docx

Title: A systematic review of methodological approaches for evaluating real-world effectiveness of COVID-19 vaccines: advising resource-constrained settings

Authors:
Dr Yot Teerawattananon, PhD
Yot.t@hitap.net
Health Intervention and Technology Assessment Program, Ministry of Public Health, Thailand
Saw Swee Hock School of Public Health, National University of Singapore, Singapore

Dr Thunyarat Anothaisintawee, PhD
Thunyarata.a@hitap.net
Department of Family medicine, Faculty of Medicine, Ramathibodi Hospital, Mahidol University, Bangkok, Thailand

Ms Chatkamol Pheerapanyawaranun, MSc
Chatkamol.p@hitap.net
Health Intervention and Technology Assessment Program, Ministry of Public Health, Thailand

Ms Siobhan Botwright, MA *(corresponding author)*
Siobhan.b@hitap.net
Health Intervention and Technology Assessment Program, Ministry of Public Health, Thailand

Dr Katika Akksilp, MD
Katika.a@hitap.net
Health Intervention and Technology Assessment Program, Ministry of Public Health, Thailand

Dr Natchalaikorn Sirichumroonwit, MD
Natchalaikorn.s@gmail.com
Health Intervention and Technology Assessment Program, Ministry of Public Health, Thailand

Dr Wanrudee Isaranuwatchai, PhD
Wanrudee.i@hitap.net
Health Intervention and Technology Assessment Program, Ministry of Public Health, Thailand

Corresponding author:

Ms Siobhan Botwright, MA

Health Intervention and Technology Assessment Program, Ministry of Public Health, Tiwanon Rd., Muang, Nonthaburi 11000, Thailand

siobhan.b@hitap.net

+662-590-4549

SUMMARY

Background

Real-world effectiveness studies are important for monitoring performance of COVID-19 vaccination programmes and informing COVID-19 prevention and control policies. We aimed to synthesise methodological approaches used in COVID-19 vaccine effectiveness studies, in order to evaluate which approaches are most appropriate to implement in low- and middle-income countries (LMICs).

Methods

For this rapid systematic review, we searched PubMed and Scopus for articles published from inception to July 7, 2021, without language restrictions. We included any type of peer-reviewed observational study measuring COVID-19 vaccine effectiveness, for any population. We excluded randomised control trials and modelling studies. All data used in the analysis were extracted from included papers. We used a standardised data extraction form, modified from STrengthening the Reporting of OBservational studies in Epidemiology (STROBE). Study quality was assessed using the REal Life EVidence AssessmeNt Tool (RELEVANT) tool. This study is registered with PROSPERO, CRD42021264658.

Findings

Our search identified 3,214 studies, of which 26 were eligible for analysis. All studies were conducted in 7 high-income countries and the majority assessed mRNA vaccines (81% mRNA only, 15% mRNA and viral vector). Twenty-one of the 26 studies (81%) used a cohort study design. There was significant heterogeneity for full vaccination effectiveness estimates across studies (infection: n=17, mean=79%; hospitalisation: n=7, mean=89%; death: n=3, mean=92%). Follow-up time for all studies was short (mean=9.5 weeks). Across studies, short follow-up time and limited assessment and mitigation of potential confounders, including previous SARS-CoV-2 infection and healthcare seeking behaviour, were major limitations.

Interpretation

This review summarises methodological approaches for evaluating real-world effectiveness of COVID-19 vaccines and highlights the lack of such studies in LMICs, as well as the importance of context-specific vaccine effectiveness data. Further research in LMICs will refine guidance for conducting real-world COVID-19 vaccine effectiveness studies in resource-constrained settings.

Funding

Health Systems Research Institute.

INTRODUCTION

The COVID-19 pandemic has placed a significant toll on health systems and economies. With the development and roll-out of COVID-19 vaccines, policymakers in low- and middle-income countries (LMICs) now have an additional tool to control the pandemic, with the potential to ease lockdowns and other non-pharmaceutical interventions. Yet there is increasing evidence to suggest that vaccines are not a magic bullet, and policymakers will have to identify how to best use vaccines as part of a comprehensive set of interventions[1]. In the immediate term, vaccination programme constraints, both in terms of vaccine supply as well as the capacity of health programmes to deliver vaccine at an unprecedented scale, mean that policymakers must identify how best to target vaccines for greatest impact. In the longer-term, financial sustainability is likely to become an ever more pressing issue. Policymakers have been able to allocate emergency funding to finance COVID-19 prevention and control measures, and many financial institutions have unlocked access to grants and concessional loans to tackle the pandemic[2]. However, as more data become available on vaccine duration of protection, protection against transmission, and protection against COVID-19 variants, policymakers will have to decide which vaccination strategies are sustainable and most appropriate to implement in their context[3]. Already there are stark differences in COVID-19 vaccination coverage targets between countries, ranging from those aiming to vaccinate 30% of the population to those aiming for full population coverage[4].

To inform evidence-based policies on the rational use of COVID-19 vaccines, LMICs require real-world data on the effectiveness of vaccines in their context. Efficacy data from clinical trials are important for regulatory authorities to identify if a vaccine works and if it is safe. However, there are a number of limitations in using efficacy data for policy. Firstly, clinical trials use strict inclusion and exclusion criteria, which are not necessarily representative of all eligible populations for vaccination[5-7]. For COVID-19, a number of vaccines have been recommended for use with limited data on effectiveness in the elderly, pregnant women, and populations with comorbidities, despite these being priority target groups in many countries[8-11]. Second, the setting of clinical trials may not reflect local epidemiology. COVID-19 vaccine clinical trials have been conducted in settings with different circulating strains, diverse underlying population health, varying transmission dynamics and non-pharmaceutical interventions (NPIs), and measuring different outcomes[12]. Finally, due to their nature, efficacy studies are unable to address programmatic issues around health service utilization or off-label use[5]. For COVID-19 vaccines, this includes issues such as timely receipt of the second dose, modified vaccine schedules to address supply shortages or to align timing across vaccine products, vaccine acceptance and hesitancy (especially among specific population groups), interchangeability for mixed product schedules, cold chain excursions and other logistics issues, among others[13].

Real-world effectiveness studies are important for informing policy decisions, as an estimate of the context-specific performance of vaccines[13]. The results from real-world effectiveness studies not only monitor impact, but also give country-specific inputs for modelling future strategies for vaccination and relaxation of NPIs, as well as justifying budget allocation into, or away from, the COVID-19 vaccination programme. Due to the nature of real-world effectiveness studies, they can be subject to selection bias, confounding factors, and missing data, therefore requiring careful study design[5,14,15]. The World Health Organization (WHO) has published an interim guidance for conducting vaccine effectiveness studies in LMICs[13], and is maintaining a landscape of observational study designs for COVID-19 vaccination effectiveness[16]. However, to our knowledge, there is no systematic review of published real-world effectiveness study designs for COVID-19 vaccination, to support LMICs to understand which study designs are most feasible to implement in their settings, and the advantages and drawbacks of different approaches. This review was commissioned by the Thai government to summarise methodological approaches being used to study real-world COVID-19 vaccine effectiveness, to assess the quality of published literature, and to consider which best-practice approaches are most suitable for implementation in Thailand and other LMICs.

METHODS

Search strategy and selection criteria

We conducted a systematic review of the literature to identify peer-reviewed research studies on COVID-19 vaccine effectiveness, in order to analyse the study design and methods for applicability to LMICs. We chose a rapid review methodology as a streamlined approach to quickly inform policymakers and researchers in Thailand and other LMICs that are in the process of developing vaccine effectiveness studies. Since the objective of the review was to analyse methodological approaches, we did not conduct meta-analysis to summarise the results.

We included research studies published in academic journals in any language, which reported on the effectiveness of COVID-19 vaccination in real-world settings. We therefore included any type of observational study, including cohort studies (prospective and retrospective), case control studies, test-negative design case-control studies, and screening studies, but excluded randomised control trials (RCTs) and modelling studies. Primary research articles were eligible, as were letters to the editor, correspondence, reports, or rapid communications, provided that the methods were adequately described for data extraction and quality assessment of study design. Due to our focus on methodological approaches, we only included peer-reviewed literature, as quality assurance for study design and reporting. We did not exclude studies based on population of interest, but restricted inclusion to studies measuring the following outcomes: asymptomatic SARS-CoV-2 infection, symptomatic SARS-CoV-2 infection, severe SARS-CoV-2 infection (as measured by hospital admission, ICU admission, or clinical diagnosis), or death from SARS-CoV-2 infection.

We executed a search strategy (Appendix) of articles published from inception to July 7, 2021, in the MEDLINE (via PubMed) and Scopus databases. Search terms were constructed according to intervention of interest (COVID-19 vaccine) and study design (e.g. cohort study, post-marketing study, effectiveness analysis). Reference lists of the included studies were searched to identify additional relevant studies. In the first stage, titles and abstracts were screened independently by two reviewers, each from one of two separate teams. Any disagreement was resolved by YT or TA. In the second stage, full text was reviewed for inclusion/exclusion by a single reviewer.

Data analysis

All authors extracted data using a structured form modified from STrengthening the Reporting of OBservational studies in Epidemiology (STROBE), the reporting standard for observational studies[17]. Data were abstracted on study characteristics (objectives, type of study design, country, study duration, funding source); study sample (population, sample size, presence of variants of concern); intervention (partial or full vaccination, vaccine product received); study outcomes; data collection and measurement methods (including utilisation of existing database); data analysis methods (subgroup analysis, statistical model, sensitivity analysis, management of missing data and potential confounders); results (by outcome of interest); study limitations; and ethical approval and/or consent requirements. Type of study design was classified by the authors based on definitions from the WHO interim guidance on evaluation of COVID-19 vaccine effectiveness[13]. For the results, vaccine effectiveness (%) by outcome was recorded. For studies reporting incidence rate ratio (IRR), the formula (1-IRR)*100 was used to calculate vaccine effectiveness. If effectiveness data were unclear, the study was not included in the comparison of effectiveness but was kept for the qualitative analysis of study design and methods. The quality of studies was assessed by two independent reviewers using the REal Life EVidence AssessmeNt Tool (RELEVANT) tool[18]. Each primary and secondary sub-item was scored as 1 (yes) if performed or reported in the study, otherwise a score of 0 (no) was assigned. YT and TA resolved any discrepancy in scoring. Qualitative analysis identified areas of limited evidence and highlighted opportunities to strengthen COVID-19 vaccine effectiveness study methodology.

Figures were produced using R, version 4.1.0 (Camp Pontanezen). The review protocol is registered at PROSPERO, CRD42021264658.

Role of the funding source
This study was funded by the Health Systems Research Institute, grant number 64134002RM011L0. The funder of the study had no role in study design, data collection, data analysis, data interpretation, or writing of the report.

RESULTS

We identified 3,214 articles through the database search. No additional articles were identified from searching reference lists. After removal of duplicates (497) and exclusion of studies based on screening the abstract (2,659) or the full text (32), 26 studies were included in this qualitative synthesis (Figure 1). Of the 32 studies excluded during full text screening, 27 reported on an excluded outcome (not effectiveness) and 5 were an excluded study type (randomised control trial or modelling study). All studies were in English, except one study in Spanish. Table 1 summarises study characteristics.

All 26 studies identified were published in 2021 and conducted in 7 high-income countries (HICs) (Table 2). No studies were identified from Africa or Asia. Presence of circulating variants were reported in 8 (31%) studies; the only variant of concern (VOC) mentioned was the alpha variant, reported in 7 studies[11,19,23,28,29,33,38]. Most studies assessed effectiveness of mRNA vaccines (21 studies), followed by an mRNA and a viral vector vaccine (4 studies), and 1 study for an inactivated vaccine. Ethical approval was required in 17 studies (65%), with 8 studies (31%) not reporting on ethical approval. Most studies (16, 61%) did not report on funding source; for the other studies, 5 (19%) were publicly funded, 2 (8%) funded through public and private funds, 1 (4%) through not-for-profit private funding, and 2 (8%) did not receive funding.

Most studies (20 of 26, 77%) reported on vaccine effectiveness against either COVID-19 infection, hospitalisation, or death, whereas 2 studies reported 2 outcomes (hospitalisation and infection[29], hospitalisation and death[37]) and 4 studies reported on all 3 outcomes[19,22,27,41]. For confirmation of COVID-19 infection, 24 studies confirmed diagnosis with reverse transcription polymerase chain reaction (RT-PCR) and 2 studies used RT-PCR as the main method of confirming diagnosis, but either allowed rapid antigen test for symptomatic cases [29] or if RT-PCR was not available [41].

Of the studies measuring vaccine effectiveness against infection, 19 are cohort studies, 3 test-negative design case control studies, and 1 screening method (Figure 2). The most common study type is retrospective cohort study using immunisation registries and medical databases (11 studies). Only three studies considered asymptomatic infection among patients under investigation, healthcare workers and randomly selected individuals in the community [11,29,38]. Most cohort studies were conducted among healthcare workers undergoing routine RT-PCR testing as part of the hospital surveillance system. Sample size ranged from 189 to 10,187,720 (mean 773,736; median 9,000). For vaccine effectiveness against hospitalisation and/or death, we identified 7 cohort and 2 test negative design case control studies. Contrary to infection studies, none had healthcare workers as the population. All studies in the general population used national level surveillance data. Sample size ranged from 189 to 10,187,720 (mean 2,709,298; median 675,083). The test negative designs had small sample sizes compared to cohort studies.

Comparison of effectiveness estimates across studies shows significant heterogeneity in results (Figure 3). Effectiveness of partial vaccination ranged from 16% to 84% (mean 61.3%) against infection, 37% to 91% (mean 70.0%) against hospitalisation, and 46% to 55% (mean 50.7%) against death. For full immunisation, vaccine

effectiveness ranged from 61% to 95% (mean 78.6%) against infection, 72% to 97% (mean 88.7%) against hospitalisation, and 86% to 97% (mean 92.4%) against death. Overall, vaccine effectiveness against infection showed wide variation even for the same vaccine type, whereas effectiveness against hospitalisation and death was more uniform across vaccine type and setting.

Looking across study types, cohort studies generally had a lower quality than other study designs (Appendix). Only 6 of the 26 studies reported registration or publication of the study protocol and less than half (11 of 26) reported on potential conflicts of interest (Figure 4). Regarding study methods, there were a number of limitations across studies. Firstly, due to the short time since vaccine roll-out, follow-up time for all studies was very short (mean 6.2 weeks for studies with infection outcomes, 10.3 weeks for hospitalisation or death outcomes). For the primary analysis, 20 studies followed best practice and only included outcomes occurring more than 14 days after first dose or at least 7 days after second dose of vaccination; 3 studies included outcomes from 12 days after the first dose[10,30,40]; 1 study measured from 6 days after the second dose[9]; and 2 studies assessed outcomes occurring any time after immunisation[24,35]. Secondly, although, as observational studies, many studies aimed to include as many participants as possible, only 7 studies reported calculating a sample size a priori (Figure 4); all studies that did so were cohort studies with infection as the outcome of interest. Thirdly, most studies did not show inclusion/exclusion of study participants as a flowchart, although all studies were judged to be in a relevant population and setting. Only 11 studies reported on vaccine coverage during the study period. For the test-negative design case control studies, the 2 studies looking at hospitalisation or death were conducted in older adults, whilst 2 of the 3 studies measuring infection rates were conducted in health workers (Figure 2). However, 1 test-negative design case control study was in the general population, which may be subject to collider bias. Fourthly, due to the observational study design, selection bias and confounding effects were inevitable limitations, and 8 studies lacked explicit assessment and mitigation of potential confounders (Figure 4). Covariates reported included age (16 studies), sex (14 studies), socio-demographic factors (ethnicity/religion) (9 studies), socio-economic status (7 studies), and chronic conditions (5 studies). Healthcare seeking behaviour based on vaccination status was measured in 7 studies. No study in our review measured adherence to NPIs and none of the test-negative design studies measured chronic disease status or respiratory viral infection. Previous SARS-CoV-2 infection was not measured in 18 studies, participants with prior infection were excluded in 7 studies, and 1 study included prior infection in sensitivity analysis. No study reported percentage of COVID-19 deaths in the vaccinated non-study population to prevent survivorship bias. Misclassification of outcomes was mentioned as a limitation in 2 test-negative design case control studies [19,21] and 9 cohort studies [11,22,25-27,31,34,39,41]. Finally, only 10 of 26 studies reported on the extent of missing data (Figure 4).

DISCUSSION

To our knowledge, this is the first systematic review of methodologies for COVID-19 vaccine effectiveness studies. Given the scale of COVID-19 vaccine roll-out thus far, our review identified relatively few studies assessing real-world vaccine effectiveness. Of the existing studies, we identified significant heterogeneity in estimates of vaccine effectiveness, likely due to differences in population groups and outcomes studied, study design, and presence of VOCs. All studies identified are from HICs, often utilising national databases (which may not exist or may be of poorer quality in LMICs), and the great majority assessed mRNA vaccines, which are more prevalent in HICs but only represent a third of the vaccines with WHO Emergency Use Listing (EUL)[42] and one-fifth of COVAX secured supply from legally binding agreements[43]. Whilst the WHO landscape of observational studies has identified pre-prints and registered studies being conducted in six middle income countries (Argentina, Brazil, India, Indonesia, Tunisia, Turkey)[16], between our review and the WHO landscape document there are no real-world effectiveness studies for half of the vaccines that have received WHO EUL and no study in low-income countries. These findings

underscore the importance of advocating for real-world effectiveness studies on all approved COVID-19 vaccines and across diverse LMIC settings.

Our review has highlighted several important components to consider at the outset of designing a real-world effectiveness study of COVID-19 vaccines, including the appropriate study design, study population, outcome, and time for follow-up. The most common study design identified in our review was a cohort approach, which may have been facilitated by the presence of large, reliable, and inter-linked databases in study countries. Test negative design case control studies were the second most common study design, but we did not identify any case-control studies in this review. We hypothesise that this finding may be because of the challenges in enrolling an unbiased comparison group: the low number of case-control registered studies and pre-prints suggests that we did not select against case-control studies by restricting our search to peer-reviewed articles[16].

In studies assessing symptomatic or asymptomatic infection as an outcome, healthcare workers were the most common study population. In many studies, healthcare workers were an opportune population due to routine symptomatic or RT-PCR screening activities undertaken within the health system. Conversely, we identified no studies using healthcare workers as the study population for the outcomes hospitalisation and death, which we hypothesise as being due to the low number of severe outcomes in this group[44]. Instead, studies either selected populations at high risk of disease (such as the elderly) or utilised large national databases to assess outcomes in the general population. If large-scale studies are not feasible, or rely on poor-quality databases, LMICs may find that test-negative designs are most feasible to implement, as recommended by the WHO interim guidance[13]. Regarding study population and outcome, we suggest that health workers may be the most appropriate population for studies measuring effectiveness against infection, whereas studies on hospitalisation/death may best focus on elderly populations or other high risk groups.

Given the short timeline since COVID-19 vaccine introduction, the duration of all studies was less than five months. As would be expected, studies looking at hospitalisation and death tended to have longer duration than those assessing infection. However, the short follow-up time may have underestimated vaccine effectiveness against severe outcomes, and means that studies were not able to consider duration of protection, which will be important in informing strategies for delivering booster doses among different populations. Studies of longer duration may also allow assessment of changing vaccine effectiveness with the emergence of new VOCs. Despite widespread concern on protection of COVID-19 vaccines against VOCs, many studies did not assess prevalence of variants and none reported on the delta strain. The WHO landscape of observational studies for vaccine effectiveness suggests that this is likely to remain a significant gap in the literature for future research to consider: only three pre-prints from HICs report on the delta variant and only four registered studies, all in HICs, will assess variants[16]

Our review highlights several gaps that merit further study, alongside opportunities to strengthen the quality of real-world vaccine effectiveness studies. Firstly, we identified a need for studies in LMICs, especially in Africa and Asia, as well as effectiveness studies with a longer duration and covering all vaccines with WHO EUL. Without information on vaccine effectiveness for all licensed products, governments may face diminishing public confidence towards the vaccines in use in their country. Second, most studies did not calculate (or report) the sample size a priori. Since many LMICs are unlikely to be able to replicate the large-scale studies from HICs, calculating minimum sample size will be very important, and should account for differences in access to healthcare services and health seeking behaviour in LMICs, as compared with HICs. Third, we identified weaknesses across studies in identifying and mitigating against potential confounders, and in reporting on missing data. Missing data are likely to be a greater issue in LMICs and differences in healthcare utilisation are likely to be more pronounced than in many HICs, requiring a well-considered plan for identifying and dealing with confounders and missing data. In particular, we note that many studies either did not measure for previous SARS-CoV-2 infection or used this as an exclusion

criterion. If the infrastructure exists, we recommend testing for previous infection and conducting sensitivity analysis including this group, to avoid selecting the sample based on exposure risk. Finally, most studies failed to report on the presence of VOCs or on conflict of interest, including funding source. The former is important to respond to changes in vaccine effectiveness with new variants, and the latter is important for credibility of studies for policymaking. Accordingly, we recommend a number of additions to the WHO interim guidance on evaluation of COVID-19 vaccine effectiveness. The document would benefit from further guidance on setting an appropriate time horizon for studies, alongside guidance on designing studies that can be conducted with limited resources. We also propose the inclusion of practical guidance on identifying important confounders for a given setting and management of missing data. Finally, we suggest the inclusion of managing and reporting conflict of interest, as a fundamental part of study design.

There are several limitations to our review. We conducted the review only seven months after the first COVID-19 vaccines were licensed, limiting the number of studies and timeframe, as well as skewing our search results towards HICs, which were the first to introduce COVID-19 vaccination. Restricting our search to peer-reviewed articles further limited the number of results and favoured earlier studies in HICs with limited outcomes based on available data. Because of these limitations, our review was unable to objectively compare approaches that may be more appropriate to LMIC settings and yielded insufficient studies to conduct a meta-analysis. Furthermore, because of an urgent request from the Thai government, we employed rapid review methodology. Particularly for the quality assessment of studies, we had to make assumptions based on reporting in the article, whereas contacting study authors for clarifications may have yielded further information to enhance our analysis.

Despite the importance of real-world effectiveness studies for informing national COVID-19 prevention and control policies in LMICs, existing studies tend to focus on settings, available vaccines, and VOCs specific to a handful of HICs. Although WHO recommends against conducting effectiveness studies in each country[13], in light of the heterogeneity between studies, we argue that there is benefit to each country designing and conducting effectiveness studies, subject to available resources. Considerable funding has been made available from the public sector for COVID-19 vaccine development and deployment. We therefore argue that it is imperative for the public sector to continue funding to the end of the product development continuum and finance studies on effectiveness and impact, not just domestically but across countries, given the global nature of the COVID-19 pandemic.

In summary, our review highlights the importance of local vaccine effectiveness data, and suggests that test-negative case control studies with sample size calculated a priori may be most practical to implement in LMICs, especially since reliable and interlinked databases for COVID-19 vaccination, diagnosis and treatment often do not exist in these settings. We highlight the limited experience conducting vaccine effectiveness in LMICs, but emphasise the importance of such studies for policymakers in LMICs to develop and monitor vaccination policies, as well as to enhance public confidence in vaccination. We call on the global community to support LMICs to lead and implement COVID-19 vaccine effectiveness studies in their settings, as a priority research area moving forward.

Table 1. Characteristics of COVID-19 vaccine real-world effectiveness studies meeting inclusion criteria.

	Country	Funding source	Population	Sample size	Study design*	Study time frame	Database(s)	Type(s) of vaccine	Outcome	Follow-up time
Lopez-Bernal [19]	U.K.	None	Elderly people aged ≥70 years old	265,745	Test negative case-control design	October 26, 2020 - February 21, 2021	National Immunisation Management System and hospital admission data	BNT162b2, ChAdOx1-S	SAR-CoV2 infection, hospital admissions, deaths	After 1st dose, 0-3, 4-6, 7-9, 10-13, 14-20, 21-27, 28-34, 35-41 and ≥42 days.; After 2nd dose, 0-3, 4-6, 7-13, and ≥14 days.
Vasileiou [20]	U.K.	UK Research and Innovation (Medical Research Council), Research and Innovation Industrial Strategy Challenge Fund, Health Data Research UK	General population	5.4 million	Prospective cohort study	December 8, 2020 - February 22, 2021	Early Pandemic Evaluation and Enhanced Surveillance of COVID-19—EAVE II—database, Scottish Morbidity Record 01 database, and Rapid Preliminary Inpatient Data.	BNT162b2, ChAdOx1-S	Hospital admissions due to SARS-CoV-2 infection	After 1st dose, 0-6, 7-13, 14-20, 21-27, 28-34, 35-41, and ≥42 days

	Country	Funding source	Population	Sample size	Study design*	Study time frame	Database(s)	Type(s) of vaccine	Outcome	Follow-up time
Tenforde [21]	USA	Not stated	Adults with COVID-19–like illness admitted to 24 hospitals in 14 states. Patients were eligible if they were ≥65 years on the date of hospital admission, received clinical testing for SARS-CoV-2 by RT-PCR or antigen test within 10 days of illness onset, and had onset of symptoms 0–14 days before admission.	417	Observational study	January 1–March 26, 2021	Not stated	BNT162b2	SAR-CoV2 infection and hospital admissions	1) First vaccine dose <14 days before illness onset<br>2) Within 14 days prior to onset of COVID-19–like illness<br>3) Partially vaccinated, receipt of 1 dose of a 2-dose vaccine series ≥14 days before illness onset or 2 doses with the second dose received <14 days before illness onset<br>4) Fully vaccinated, defined as receipt of both doses of a 2-dose vaccine series ≥14 days before illness onset

	Country	Funding source	Population	Sample size	Study design*	Study time frame	Database(s)	Type(s) of vaccine	Outcome	Follow-up time
Haas [22]	Israel	Israel MoH and Pfizer	≥16 years old residents of Israel	Isreali population in 1 of 4 nationwide medical insurance programmes	Observational study	January 24 - April 3, 2021	Nationwide Surveillance Data	BNT162b2	SAR-CoV2 infection, hospital admissions, deaths	At least 7 days after second dose, ≥7 days after the second dose, with the medium follow-up time of 48 days
Sansone [23]	Italy	Not stated	Healthcare workers in Brescia	6,904	Observational study	January 25, 2021 - April 13, 2021	No database used	BNT162b2	SAR-CoV2 infection	At least 7 days after second dose, ≥7 days after the second dose, with the medium follow-up time of 48 days
Keehner [24]	USA	Not stated	Healthcare workers in University of California, San Diego (UCSD) and University of California, Los Angeles (UCLA)	36,659	Observational study	December 16, 2020 – February 9, 2021	Electronic employee health record system at UCSD and UCLA	BNT162b2, mRNA 1273	SAR-CoV2 infection	After 1st dose: 7 days, 14 days, 21 days, 22nd day or later, before 2nd dose<br><br>After 2nd dose: 7 days, 14 days, 15 days or later
Thompson [25]	USA	Not stated	Healthcare workers, first responders, and frontline workers	3,950	Observational study	December 14–18, 2020 - March 13, 2021.	No database used	BNT162b2, mRNA 1273	SAR-CoV2 infection	Partially immunized: ≥14 days after receiving first dose only, ≥14 days after first dose through

	Country	Funding source	Population	Sample size	Study design*	Study time frame	Database(s)	Type(s) of vaccine	Outcome	Follow-up time
										receipt of second dose<br><br>Fully immunized: ≥14 days after second dose
Fabiani [26]	Italy	Not stated	Frontline healthcare workers	6,423	Retrospective cohort study	December 27, 2020 - March 24, 2021	Local COVID-19 surveillance database	BNT162b2	SAR-CoV2 infection	21 days after 1st dose; 7 days after 2nd dose
Cavanaugh [27]	USA	Not stated	Residents and healthcare workers	189	Retrospective cohort study	January 10 - March 1, 2021	Immunization registry review; facility interviews; medical records reviews	BNT162b2	SAR-CoV2 infection, symptomatic COVID-19 cases, hospital admissions, deaths	14 days after 2nd dose
Hall [11]	U.K.	Public Health England, UK Department of Health and Social Care, and the National Institute for Health Research	Healthcare workers and staff ≥18 years old	23,324	Prospective cohort study	Dec 7, 2020 - Feb 5, 2021	Participants enrolling to the National Immunization Management System	BNT162b2	SAR-CoV2 infection	For outcome: 8 weeks after first dose<br><br>For vaccine: 21 days after 1st dose; 7 days after 2nd dose
Benenson [28]	Israel	Not stated	Healthcare workers	6,680	Descriptive cohort study	8 weeks after Dec 20, 2020	Not stated	BNT162b2	SAR-CoV2 infection	8 weeks after first dose (Dec 20, 2020)

	Country	Funding source	Population	Sample size	Study design*	Study time frame	Database(s)	Type(s) of vaccine	Outcome	Follow-up time
Martínez-Baz [29]	Spain	The Horizon 2020 program of the European Commission and the Carlos III Institute of Health with the European Regional Development Fund	Individuals aged ≥18 years covered by the Navarre Health Service with close contacts of laboratory-confirmed COVID-19 cases	20,961	Prospective cohort study	January to April 2021	Not stated	BNT162b2, ChAdOx1-S	SAR-CoV2 infection	Not stated
Chodick [30]	Israel	Not stated	All Maccabi Healthcare Services (MHS) members aged 16 years or older who were vaccinated during a mass immunization program	503,875	Comparative effectiveness study	December 19, 2020 - January 15, 2021	Maccabi Healthcare Services	BNT162b2	SAR-CoV2 infection	Follow-up period for assessing VE ended in day 24 after the first dose, 3 days after day 21, at which point the second dose can be given.
Jameson [31]	USA	None	All healthcare workers in a hospital	4,318	Screening	December 17, 2020 - March 24, 2021	Not stated	BNT162b2	SAR-CoV2 infection	Not stated

	Country	Funding source	Population	Sample size	Study design*	Study time frame	Database(s)	Type(s) of vaccine	Outcome	Follow-up time
Pilishvili [9]	USA	Not stated	Routine employee testing performed based on site-specific occupational health practices.	1,843	Test negative case-control study	January–March 2021	Not stated	BNT162b2, mRNA 1273	SAR-CoV2 infection	Not stated
Daniel [32]	USA	Texas Department of State Health Services	University employees	23,234	Descriptive data report	December 15, 2020 - February 28, 2021	University of Texas Southwestern Medical Center (UTSW)	BNT162b2, mRNA 1273	Decrease in the number of employees who are either in isolation or quarantine and reduction in the incidence of infections	Not stated

	Country	Funding source	Population	Sample size	Study design*	Study time frame	Database(s)	Type(s) of vaccine	Outcome	Follow-up time
Angel [33]	Israel	Not stated	Healthcare workers	6,710	Retrospective cohort study	December 20, 2020 - February 25, 2021	Hospital data	BNT162b2	SAR-CoV2 infection	December 20, 2020, to January 2, 2021 (period 1), screened monthly or biweekly depending on their risk of exposure; from January 3 to 14, 2021 (period 2), wide screening regardless; January 15, 2021- February 25 (period 3), screen medium to high exposure risk and non–fully vaccinated health care workers screened monthly to weekly
Amit [34]	Israel	Not stated	Healthcare workers	9,109	Retrospective cohort study	December 19, 2020 - January 24, 2021	Not stated	BNT162b2	SAR-CoV2 infection	Days 1–14 and 15-28 after the first dose of the vaccine

	Country	Funding source	Population	Sample size	Study design*	Study time frame	Database(s)	Type(s) of vaccine	Outcome	Follow-up time
Amit [35]	Israel	Not applicable	Healthcare workers	4,081	Active and passive surveillance	December 2-27, 2020	Primary data conduct using by questionnaire, hotline, on-call, web-based, and laboratory-confi rmed COVID-19	BNT162b2	SAR-CoV2 infection	A week after first dose
Britton [36]	Israel	Not applicable	Skilled nurse residents	463	Retrospective cohort study	December 29, 2020 - February 12, 2021	The electronic medical record chart abstraction	BNT162b2	SAR-CoV2 infection	From >14 days after dose 1 through 7 days after dose 2
Dagan [37]	USA	Not stated	Healthcare workers	4.7 million	Retrospective observational study	December 20, 2020 - February 1, 2021	Clallit Health Services (CHS)	BNT162b2	SAR-CoV2 infection, symptomatic COVID-19 cases, severe COVID-19 cases, hospital admissions, deaths	1.5 months or the follow-up ended at the earliest of the following events: occurrence of an outcome event, death unrelated to Covid-19, vaccination (for unvaccinated controls), vaccination of the matched control (for vaccinated persons), or the end of the study period.

	Country	Funding source	Population	Sample size	Study design*	Study time frame	Database(s)	Type(s) of vaccine	Outcome	Follow-up time
Pritchard [38]	U.K.	Department of Health and Social Care, Welsh Government and Department of Health on behalf of the Northern Ireland Government and Scottish Government.	General population ≥16 years old	383,812	A large household survey with longitudinal follow-up	December 1, 2020 - May 8, 2021	The Office for National Statistics (ONS) COVID-19 Infection Survey	BNT162b2, ChAdOx1-S	SAR-CoV2 infection and infection severity	Not vaccinated; not previously positive; >21 d before vaccination, Not vaccinated; not previously positive; 1–21 d before vaccination, Vaccinated with 0–7 d ago, Vaccinated with 8–20 d ago, ≥21 d after first dose; no second dose, Post-second dose, Not vaccinated; previously positive <4 months ago, Not vaccinated; previously positive ≥4 months ago
Domi [39]	USA	Not stated	Healthcare workers from CDC Tiberius system for Long Term Care facilities	12,347	Retrospective observational study	December 20, 2020 - February 7, 2021	The CMS National Health Safety Network (NHSN) Public File Data	BNT162b2	SAR-CoV2 infection and mortality	Each week, starting 3 weeks after the first vaccination clinic took place

	Country	Funding source	Population	Sample size	Study design*	Study time frame	Database(s)	Type(s) of vaccine	Outcome	Follow-up time
Jones [40]	U.K.	Wellcome Trust/Medical Research Council/NHS Blood and Transplant/E PSRC	Healthcare workers	Approximat ely 9000	Retrospective cohort study	January 18, 2021 - January 31, 2021	Hospital-laboratory interface software, Epic (Verona, WI)	BNT162b2	SAR-CoV2 infection	Not clearly defined
Gras-Valenti [10]	Spain	Not stated	Healthcare workers in Alicante General Hospital	268	Test negative case control	January 25, 2021 - February 7 2021	Registro Nominal de Vacunas de la Generalitat Valenciana	BNT162b2	SAR-CoV2 infection, symptomatic COVID-19 cases,	Classed as vaccinated 12 days after onset of symptoms or positive PCR for asymptomatic cases
Jara [41]	Chile	The Agency Nacional de Investigacion & Millennium Science Initiative Program	Population ≥16 years old receiving at least 1 dose of CoronaVac	10,187,720	Prospective cohort study	February 2, 2021 - May 1, 2021	Database of Fondo Nacional de Salud (FONASA), the national public health insurance program.	CoronaVac	SAR-CoV2 infection, ICU admissions, deaths	Not clearly defined

RT-PCR – reverse transcriptase polymerase chain reaction; MoH – Ministry of Health; ICU – intensive care unit; VE – vaccine effectiveness

*As reported in the study. For the purposes of standardisation in our analysis, we re-classified the following studies (in accordance with the WHO interim guidance for conducting vaccine effectiveness studies in LMICs): Tenforde et al – test negative case control design; Haas et al – retrospective cohort study; Sansone et al – retrospective cohort study; Keehner et al – retrospective cohort study; Thompson et al – retrospective cohort study; Benenson et al – screening study; Chodick et al – retrospective cohort study; Jameson et al – retrospective cohort study; Daniel et al – retrospective cohort study; Amit et al – retrospective cohort study; Dagan et al – prospective cohort study; Pritchard et al – prospective cohort study.

Table 2. General characteristics of articles on real-world effectiveness of COVID-19 vaccines

Characteristics	N (%)
**Publication year**	
2021	26 (100%)
**Publication type**	
Correspondence	2 (8%)
Letter	1 (4%)
Original (primary) research	19 (72%)
Rapid communication	2 (8%)
Report (e.g. MMWR)	2 (8%)
**Country**	
Chile	1 (4%)
Israel	7 (27%)
Italy	2 (8%)
Scotland	1 (4%)
Spain	2 (8%)
United Kingdom	4 (15%)
United States	9 (34%)
**Vaccine types**	
mRNA (BNT162b2)	16 (61%)
mRNA (BNT162b2 and mRNA-1273)	5 (19%)
mRNA and viral vector (BNT162b2 and ChAdOx1-S)	3 (12%)
mRNA and viral vector (BNT162b2, mRNA-1273 and ChAdOx1-S)	1 (4%)
Inactivated SARS-CoV-2 (CoronaVac)	1 (4%)
**Variants**	
Mentioned	8 (31%)
B.1.1.7 (alpha)	6
B.1.1.7 and B.1.525	1
R.1 lineage	1
Not mentioned	18 (69%)
**Ethical approval**	
Yes	17 (65%)
Exempted	1 (4%)
Not stated	8 (31%)
**Informed consent**	
Yes	2 (8%)
Exempted	3 (11%)
Full ethical review was not necessary	2 (8%)
Not stated	19 (73%)
**Study design**	
Test-negative design case control study	4 (15%)
Prospective cohort study	6 (23%)
Retrospective cohort study	15 (58%)
Screening methods	1 (4%)
**Outcomes (a study can have more than one outcome)**	
Infections	22
Hospitalizations	8
Mortality	6
**Financial source**	
Public	5 (19%)
Public and Private	2 (8%)
Private not for profit	1 (4%)
None	2 (8%)
Not reported	16 (61%)

MMWR - Morbidity and Mortality Weekly Report

Figure 1. Study profile.

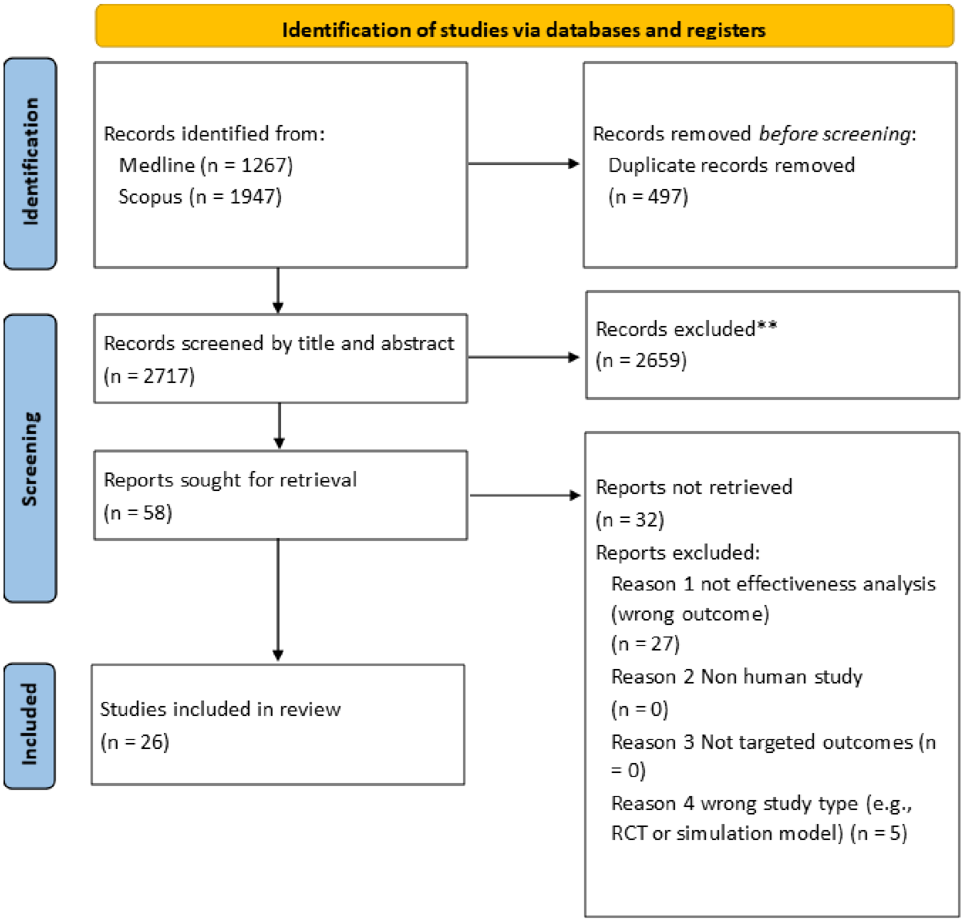

Figure 2. Study design by outcome for COVID-19 vaccine effectiveness studies meeting inclusion criteria.

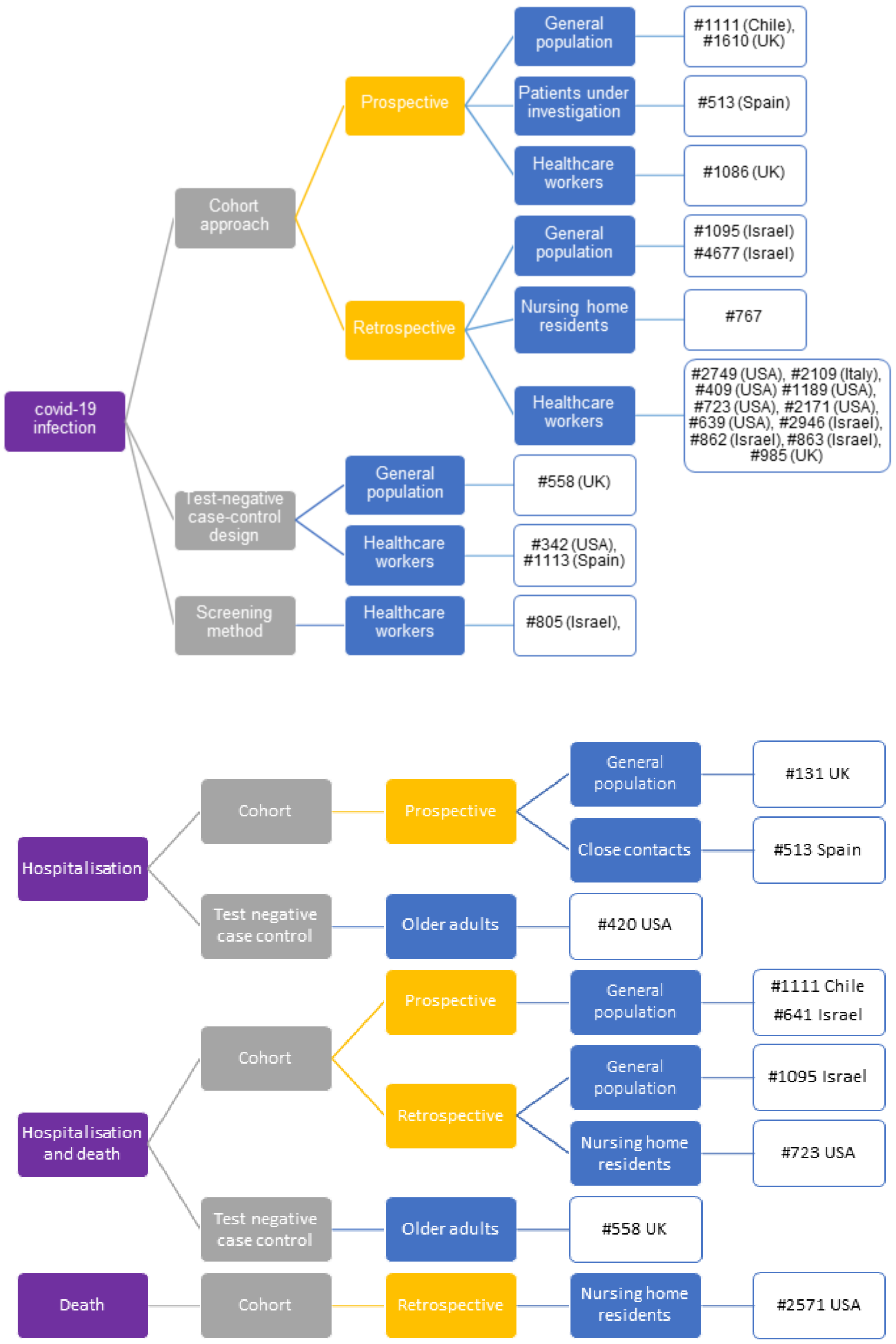

Figure 3. COVID-19 vaccine effectiveness for full vaccination (top panel) and partial vaccination (bottom panel) among included studies.

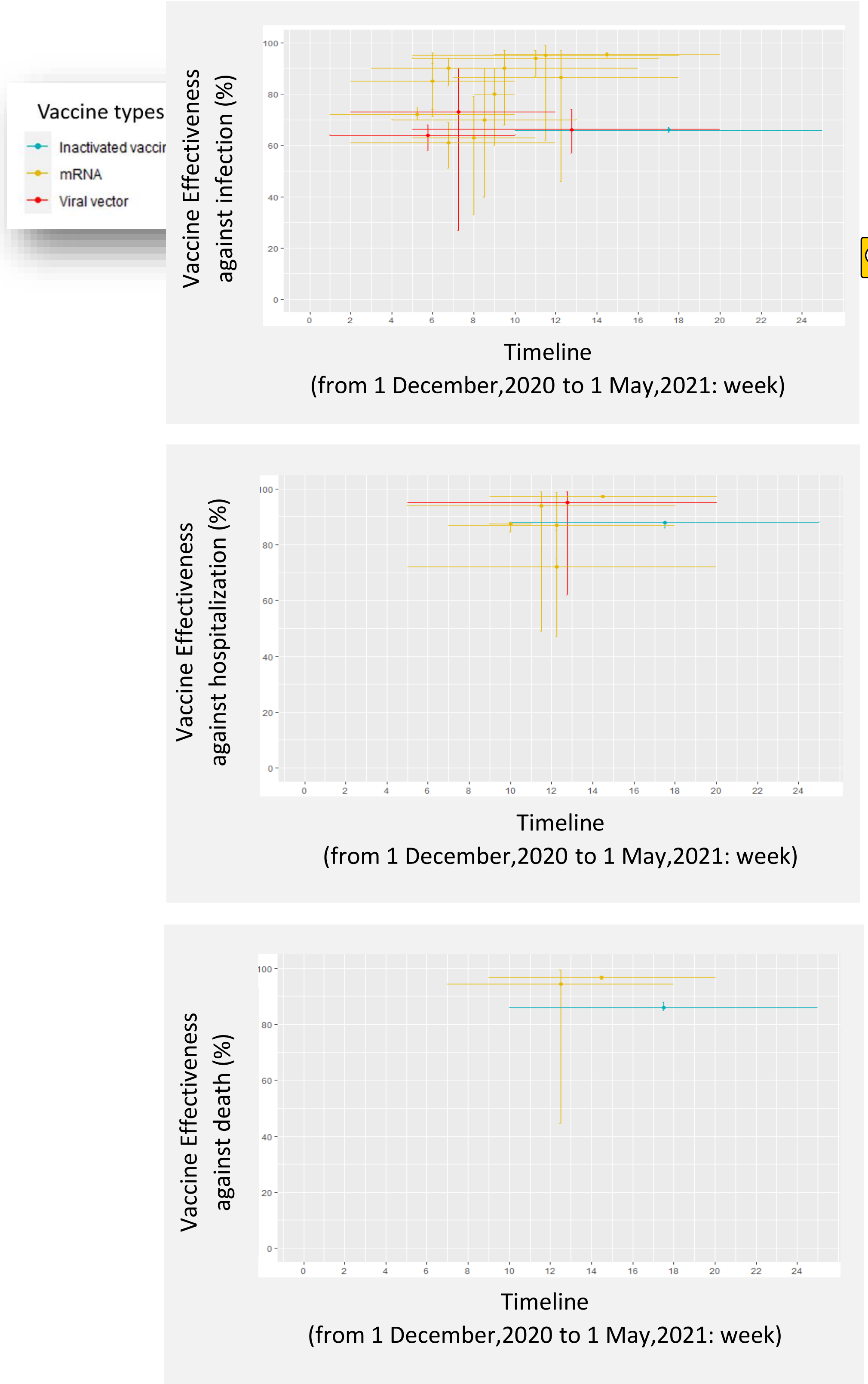

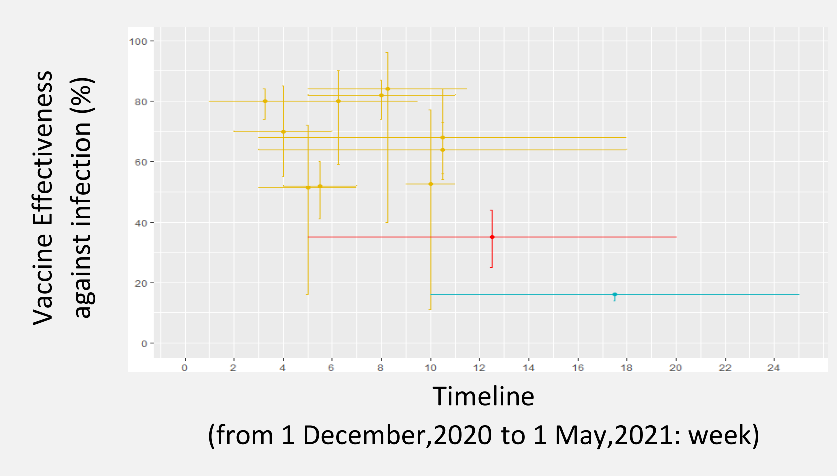
Vaccine Effectiveness
against infection (%)
100
80
60
40
20
0
0 2 4 6 8 10 12 14 16 18 20 22 24
Timeline
(from 1 December,2020 to 1 May,2021: week)

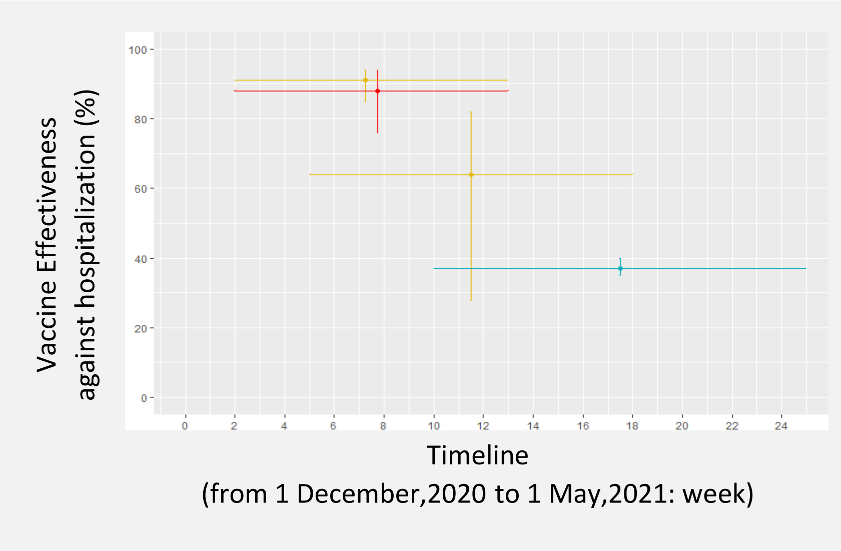
Vaccine Effectiveness
against hospitalization (%)
100
80
60
40
20
0
0 2 4 6 8 10 12 14 16 18 20 22 24
Timeline
(from 1 December,2020 to 1 May,2021: week)

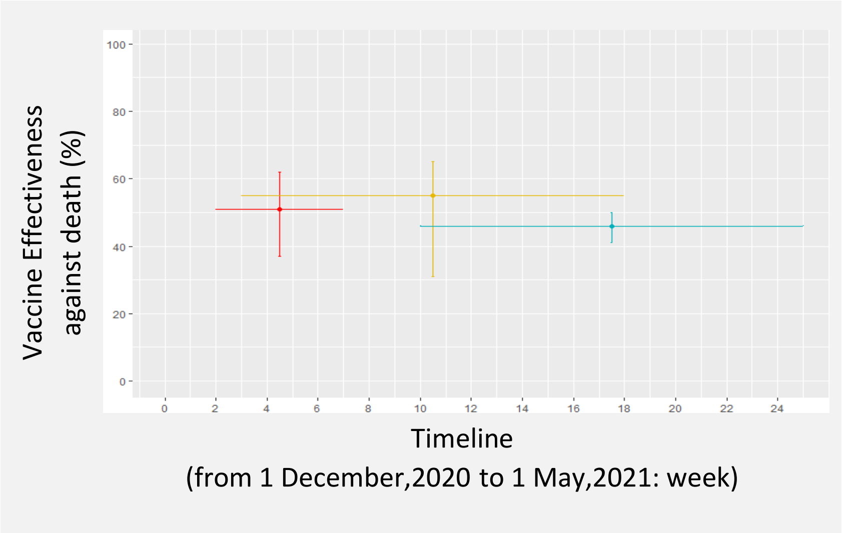
Vaccine Effectiveness
against death (%)
100
80
60
40
20
0
0 2 4 6 8 10 12 14 16 18 20 22 24
Timeline
(from 1 December,2020 to 1 May,2021: week)

Figure 4. Quality assessment using the REal Life EVidence AssessmeNt Tool (RELEVANT).

	Lopez-Bernal et al., 2021	Vasileiou et al., 2021	Tenforde et al., 2021	Haas et al., 2021	Sansone et al., 2021	Keehner et al., 2021	Thompson et al., 2021	Fabiani et al., 2021	Cavanaugh et al., 2021	Hall et al., 2021	Benenson et al., 2021	Martinez-Baz et al., 2021	Chodick et al., 2021	Jameson et al., 2021	Pilishvili et al., 2021	Daniel et al., 2021	Angel et al., 2021	Amit et al., 2021	Amit et al., 2021	Britton et al., 2021	Dagan et al., 2021	Pritchard et al., 2021	Domi et al., 2021	Jones et al., 2021	Gras-Velenti et al., 2021	Jara et al., 2021
**Clear and specific research question**	+	+	+	+	+	-	-	+	+	+	+	+	+	+	+	-	+	+	-	+	+	+	+	+	+	+
**Relevant population and setting**	+	+	+	+	+	+	+	+	+	+	+	+	+	+	+	+	+	+	+	+	+	+	+	+	+	+
Relevant interventions and outcomes are included	+	+	+	+	+	+	+	+	+	+	+	+	+	-	+	+	+	+	+	+	+	+	+	+	+	+
Protocol registration or publication	-	+	-	-	-	-	+	-	+	+	-	-	-	-	-	-	-	-	-	-	+	+	-	-	-	-
Well-described inclusion and exclusion criteria, reflecting target patients' characteristics in the real world	+	+	+	+	-	-	+	+	+	+	-	+	+	-	+	+	+	-	+	+	+	+	-	-	+	+
**Comparison groups justified**	+	+	+	+	+	-	+	+	+	+	+	-	+	+	+	+	+	+	na	+	+	+	+	+	+	+
Data sources are sufficient to support the study	+	+	+	+	+	+	+	+	+	+	+	+	+	+	+	+	+	+	-	+	+	+	+	+	+	+
High quality databases	+	+	+	+	na	+	na	uc	uc	+	uc	+	+	+	uc	na	+	na	-	+	+	+	+	+	uc	+
**Was exposure clearly defined, measured and (relevance) justified**	+	+	+	+	-	-	+	+	+	+	+	+	+	+	+	+	+	-	+	+	+	+	-	+	+	+
**Primary outcomes defined, measured and (relevance) justified**	+	+	+	+	+	+	+	+	+	+	+	+	+	+	+	-	+	+	+	+	+	+	+	+	+	+
Sufficient follow up duration	-	-	-	-	-	-	-	+	+	+	+	-	-	-	na	-	-	-	-	+	-	+	-	-	-	-
Sample size: calculated based on clear a priority hypotheses	+	-	-	-	-	-	+	+	+	+	-	-	+	-	-	-	-	-	-	+	-	-	-	-	-	-
**Thorough assessment of and mitigation strategy for potential confounders**	+	+	+	+	-	-	-	+	uc	+	-	-	+	-	+	-	+	-	-	+	+	+	-	-	+	+
**Study groups are compared at baseline**	+	+	+	-	-	-	-	+	+	+	-	-	+	-	+	+	+	-	-	+	+	+	-	-	+	+
Analyses of subgroups or integration effects reported	+	+	+	+	-	-	+	+	+	+	+	+	+	-	+	-	+	+	-	+	+	+	+	-	+	+
Sensitivity analyses performed	+	+	+	+	-	-	+	+	+	+	+	+	+	-	+	-	+	-	-	+	+	+	-	-	-	+
**Extensive presentation results**	+	+	+	+	-	-	+	+	+	+	-	+	+	-	+	-	+	-	+	+	+	+	-	-	+	+
Were confounder-adjusted estimates of treatment effects reported	+	+	-	+	-	-	+	+	-	+	-	+	+	-	+	-	+	+	-	+	+	+	+	-	+	+
Flow chart explaining all exclusions and individuals screened or selected at each stage of defining the final sample	-	-	-	-	-	-	+	-	-	-	-	-	+	-	-	-	+	-	-	+	+	-	-	-	-	+
Was follow-up similar or accounted for between groups	+	+	+	+	+	-	+	+	+	+	+	+	+	-	na	+	+	+	+	+	+	+	-	+	+	+
Did the authors describe the statistical uncertainty of their findings	+	+	+	+	-	-	+	+	+	+	-	+	+	-	+	+	+	+	-	+	+	+	-	+	+	+
Was the extent of missing data reported	+	+	+	-	-	-	+	-	-	-	-	-	+	-	+	-	+	-	-	+	+	-	+	-	-	-
**Results consistent with known information of if not, was an explanation provided**	+	+	+	+	+	+	+	+	+	+	+	+	+	+	+	+	+	+	+	+	+	+	+	+	+	+
**Are the observed treatment effects consider clinically meaningful**	+	+	+	+	+	+	+	+	+	+	+	+	+	+	+	+	+	+	na	+	+	+	+	+	+	+
Discussion of possible biases and confounding factors	+	+	+	+	-	-	+	+	+	+	-	+	+	+	+	-	+	+	+	+	+	+	+	-	+	+
Suggestions for future research to challenge, strengthen, or extend the study results	+	+	-	+	-	-	+	-	+	+	-	-	-	-	-	-	-	+	-	-	-	+	-	-	-	+
Potential conflicts of interest, including study funding, were stated	+	+	+	+	-	-	+	+	+	+	uc	+	-	-	-	+	+	-	-	-	+	+	+	+	-	+

Contributors

YT conceptualised the study. WI and YT acquired funding. YT, TA, CP, KA, and WI wrote the initial protocol. TA conducted the search. All authors conducted analysis and validation of results, with supervision from YT, TA, and WI. SB, TA, and YT wrote the original draft. CP and NS developed visuals. YT and WI reviewed and edited the final manuscript. YT and SB provided project administration.

Declaration of interests

The authors declare no competing conflicts of interest.

Data sharing

The review protocol is registered at PROSPERO, CRD42021264658. The template data extraction form and extracted data from all included studies will be made available on request.

Acknowledgements

This study was funded by the Health Systems Research Institute, grant number 64134002RM011L0. The funder of the study had no role in study design, data collection, data analysis, data interpretation, or writing of the report. The authors would like to acknowledge Vice Public Health Minister Sopon Mekthon who commissioned this study and Ms.Nuttakarn Budtarad who help on protocol development and abstract screening.

The Health Intervention and Technology Assessment Program (HITAP) is supported by the International Decision Support Initiative (iDSI) to provide technical assistance on health intervention and technology assessment to governments in low- and middle-income countries. iDSI is funded by the Bill & Melinda Gates Foundation (OPP1202541), the UK's Department for International Development, and the Rockefeller Foundation. HITAP is also supported by the Access and Delivery Partnership, which is hosted by the United Nations Development Programme and funded by the Government of Japan.

References

1. Moore S, Hill EM, Tildesley MJ, Dyson L, Keeling MJ. Vaccination and non-pharmaceutical interventions for COVID-19: a mathematical modelling study. *Lancet Infect Dis* 2021; **21**(6): 793-802.
2. Fund IM. Policy Responses to COVID-19. Policy Tracker. 2021. https://www.imf.org/en/Topics/imf-and-covid19/Policy-Responses-to-COVID-19 (accessed 19 July 2021 2021).
3. Ledford H. Six months of COVID vaccines: what 1.7 billion doses have taught scientists. *Nature* 2021; **594**(7862): 164-7.
4. Cernuschi T OBK, Pallas S. Global C-19 Vaccination Strategy. Geneva, 2021.
5. Blonde L, Khunti K, Harris SB, Meizinger C, Skolnik NS. Interpretation and Impact of Real-World Clinical Data for the Practicing Clinician. *Adv Ther* 2018; **35**(11): 1763-74.
6. Murthy VH, Krumholz HM, Gross CP. Participation in cancer clinical trials: race-, sex-, and age-based disparities. *JAMA* 2004; **291**(22): 2720-6.
7. Rothwell PM. External validity of randomised controlled trials: "to whom do the results of this trial apply?". *Lancet* 2005; **365**(9453): 82-93.

8. COVID-19 vaccines technical documents. 2021. https://www.who.int/groups/strategic-advisory-group-of-experts-on-immunization/covid-19-materials (accessed 9 July 2021.
9. Pilishvili T, Fleming-Dutra KE, Farrar JL, et al. Interim Estimates of Vaccine Effectiveness of Pfizer-BioNTech and Moderna COVID-19 Vaccines Among Health Care Personnel - 33 U.S. Sites, January-March 2021. *MMWR Morb Mortal Wkly Rep* 2021; **70**(20): 753-8.
10. Gras-Valenti P, Chico-Sanchez P, Algado-Selles N, et al. [Effectiveness of the first dose of BNT162b2 vaccine to preventing covid-19 in healthcare personnel.]. *Rev Esp Salud Publica* 2021; **95**.
11. Hall VJ, Foulkes S, Saei A, et al. COVID-19 vaccine coverage in health-care workers in England and effectiveness of BNT162b2 mRNA vaccine against infection (SIREN): a prospective, multicentre, cohort study. *Lancet* 2021; **397**(10286): 1725-35.
12. Rapaka RR, Hammershaimb EA, Neuzil KM. Are some COVID vaccines better than others? Interpreting and comparing estimates of efficacy in trials of COVID-19 vaccines. *Clin Infect Dis* 2021.
13. Evaluation of COVID-19 vaccine effectiveness. Geneva, 2021.
14. Rochon PA, Gurwitz JH, Sykora K, et al. Reader's guide to critical appraisal of cohort studies: 1. Role and design. *BMJ* 2005; **330**(7496): 895-7.
15. Mamdani M, Sykora K, Li P, et al. Reader's guide to critical appraisal of cohort studies: 2. Assessing potential for confounding. *BMJ* 2005; **330**(7497): 960-2.
16. Organization WH. Landscape of observational study designs on the effectiveness of COVID-19 vaccination. 2021.
17. von Elm E, Altman DG, Egger M, et al. The Strengthening the Reporting of Observational Studies in Epidemiology (STROBE) statement: guidelines for reporting observational studies. *Lancet* 2007; **370**(9596): 1453-7.
18. Campbell JD, Perry R, Papadopoulos NG, et al. The REal Life EVidence AssessmeNt Tool (RELEVANT): development of a novel quality assurance asset to rate observational comparative effectiveness research studies. *Clin Transl Allergy* 2019; **9**: 21.
19. Lopez Bernal J, Andrews N, Gower C, et al. Effectiveness of the Pfizer-BioNTech and Oxford-AstraZeneca vaccines on covid-19 related symptoms, hospital admissions, and mortality in older adults in England: test negative case-control study. *BMJ* 2021; **373**: n1088.
20. Vasileiou E, Simpson CR, Shi T, et al. Interim findings from first-dose mass COVID-19 vaccination roll-out and COVID-19 hospital admissions in Scotland: a national prospective cohort study. *Lancet* 2021; **397**(10285): 1646-57.
21. Tenforde MW, Olson SM, Self WH, et al. Effectiveness of Pfizer-BioNTech and Moderna Vaccines Against COVID-19 Among Hospitalized Adults Aged >/=65 Years - United States, January-March 2021. *MMWR Morb Mortal Wkly Rep* 2021; **70**(18): 674-9.
22. Haas EJ, Angulo FJ, McLaughlin JM, et al. Impact and effectiveness of mRNA BNT162b2 vaccine against SARS-CoV-2 infections and COVID-19 cases, hospitalisations, and deaths following a nationwide vaccination campaign in Israel: an observational study using national surveillance data. *Lancet* 2021; **397**(10287): 1819-29.
23. Sansone E, Tiraboschi M, Sala E, et al. Effectiveness of BNT162b2 vaccine against the B.1.1.7 variant of SARS-CoV-2 among healthcare workers in Brescia, Italy. *J Infect* 2021; **83**(1): e17-e8.
24. Keehner J, Horton LE, Pfeffer MA, et al. SARS-CoV-2 Infection after Vaccination in Health Care Workers in California. *N Engl J Med* 2021; **384**(18): 1774-5.
25. Thompson MG, Burgess JL, Naleway AL, et al. Interim Estimates of Vaccine Effectiveness of BNT162b2 and mRNA-1273 COVID-19 Vaccines in Preventing SARS-CoV-2 Infection Among Health Care Personnel, First Responders, and Other Essential and Frontline Workers - Eight U.S. Locations, December 2020-March 2021. *MMWR Morb Mortal Wkly Rep* 2021; **70**(13): 495-500.
26. Fabiani M, Ramigni M, Gobbetto V, Mateo-Urdiales A, Pezzotti P, Piovesan C. Effectiveness of the Comirnaty (BNT162b2, BioNTech/Pfizer) vaccine in preventing SARS-CoV-2 infection among healthcare workers, Treviso province, Veneto region, Italy, 27 December 2020 to 24 March 2021. *Euro Surveill* 2021; **26**(17).
27. Cavanaugh AM, Fortier S, Lewis P, et al. COVID-19 Outbreak Associated with a SARS-CoV-2 R.1 Lineage Variant in a Skilled Nursing Facility After Vaccination Program - Kentucky, March 2021. *MMWR Morb Mortal Wkly Rep* 2021; **70**(17): 639-43.
28. Benenson S, Oster Y, Cohen MJ, Nir-Paz R. BNT162b2 mRNA Covid-19 Vaccine Effectiveness among Health Care Workers. *N Engl J Med* 2021; **384**(18): 1775-7.
29. Martinez-Baz I, Miqueleiz A, Casado I, et al. Effectiveness of COVID-19 vaccines in preventing SARS-CoV-2 infection and hospitalisation, Navarre, Spain, January to April 2021. *Euro Surveill* 2021; **26**(21).

30. Chodick G, Tene L, Patalon T, et al. Assessment of Effectiveness of 1 Dose of BNT162b2 Vaccine for SARS-CoV-2 Infection 13 to 24 Days After Immunization. *JAMA Netw Open* 2021; **4**(6): e2115985.
31. Jameson AP, Sebastian T, Jacques LR. Coronavirus disease 2019 (COVID-19) vaccination in healthcare workers: An early real-world experience. *Infect Control Hosp Epidemiol* 2021: 1-2.
32. Daniel W, Nivet M, Warner J, Podolsky DK. Early Evidence of the Effect of SARS-CoV-2 Vaccine at One Medical Center. *N Engl J Med* 2021; **384**(20): 1962-3.
33. Angel Y, Spitzer A, Henig O, et al. Association Between Vaccination With BNT162b2 and Incidence of Symptomatic and Asymptomatic SARS-CoV-2 Infections Among Health Care Workers. *JAMA* 2021; **325**(24): 2457-65.
34. Amit S, Regev-Yochay G, Afek A, Kreiss Y, Leshem E. Early rate reductions of SARS-CoV-2 infection and COVID-19 in BNT162b2 vaccine recipients. *Lancet* 2021; **397**(10277): 875-7.
35. Amit S, Beni SA, Biber A, Grinberg A, Leshem E, Regev-Yochay G. Postvaccination COVID-19 among Healthcare Workers, Israel. *Emerg Infect Dis* 2021; **27**(4): 1220-2.
36. Britton A, Jacobs Slifka KM, Edens C, et al. Effectiveness of the Pfizer-BioNTech COVID-19 Vaccine Among Residents of Two Skilled Nursing Facilities Experiencing COVID-19 Outbreaks - Connecticut, December 2020-February 2021. *MMWR Morb Mortal Wkly Rep* 2021; **70**(11): 396-401.
37. Dagan N, Barda N, Kepten E, et al. BNT162b2 mRNA Covid-19 Vaccine in a Nationwide Mass Vaccination Setting. *N Engl J Med* 2021; **384**(15): 1412-23.
38. Pritchard E, Matthews PC, Stoesser N, et al. Impact of vaccination on new SARS-CoV-2 infections in the United Kingdom. *Nat Med* 2021.
39. Domi M, Leitson M, Gifford D, Nicolaou A, Sreenivas K, Bishnoi C. The BNT162b2 vaccine is associated with lower new COVID-19 cases in nursing home residents and staff. *J Am Geriatr Soc* 2021.
40. Jones NK, Rivett L, Seaman S, et al. Single-dose BNT162b2 vaccine protects against asymptomatic SARS-CoV-2 infection. *Elife* 2021; **10**.
41. Jara A, Undurraga EA, Gonzalez C, et al. Effectiveness of an Inactivated SARS-CoV-2 Vaccine in Chile. *N Engl J Med* 2021.
42. Status of COVID-19 Vaccines within WHO EUL/PQ evaluation process. 2021. https://extranet.who.int/pqweb/key-resources/documents/status-covid-19-vaccines-within-who-eulpq-evaluation-process (accessed 20 July 2021.
43. COVAX. COVAX Global Supply Forecast. 2021. https://www.gavi.org/sites/default/files/covid/covax/COVAX%20Supply%20Forecast.pdf (accessed 19 July 2021.
44. Clark A, Jit M, Warren-Gash C, et al. Global, regional, and national estimates of the population at increased risk of severe COVID-19 due to underlying health conditions in 2020: a modelling study. *Lancet Glob Health* 2020; **8**(8): e1003-e17.

Appendix

Search terms in Medline

"COVID-19 Vaccines"[MeSH Terms] AND ("effect"[All Fields] OR "effecting"[All Fields] OR "effective"[All Fields] OR "effectively"[All Fields] OR "effectiveness"[All Fields] OR "effectivenesses"[All Fields] OR "effectives"[All Fields] OR "effectivities"[All Fields] OR "effectivity"[All Fields] OR "effects"[All Fields] OR "nationwide"[All Fields] OR "real world"[All Fields] OR "post approval"[All Fields] OR "post marketing"[All Fields] OR ("cohort studies"[MeSH Terms] OR ("cohort"[All Fields] AND "studies"[All Fields]) OR "cohort studies"[All Fields] OR "cohort"[All Fields] OR "cohort s"[All Fields] OR "cohorte"[All Fields] OR "cohorts"[All Fields]) OR "adverse event"[All Fields] OR "side effect"[All Fields])

Search terms in Scopus

((TITLE-ABS-KEY (covid-19)) OR (TITLE-ABS-KEY (sars-cov-2)) OR (TITLE-ABS-KEY ("coronavirus Disease 2019")) OR (TITLE-ABS-KEY ("Severe acute respiratory syndrome coronavirus 2 "))) AND ((TITLE-ABS-KEY (vaccine)) OR (TITLE-ABS-KEY (vaccination)) OR (TITLE-ABS-KEY (immnunization))) AND (((TITLE-ABS-KEY (effectiveness)) OR (TITLE-ABS-KEY (nationwide)) OR (TITLE-ABS-KEY ("real world"))) OR (TITLE-ABS-KEY ("post approval")) OR (TITLE-ABS-KEY ("post marketing")) OR (TITLE-ABS-KEY (cohort)) OR (TITLE-ABS-KEY ("adverse event")) OR (TITLE-ABS-KEY ("side effect")))

Studies excluded at full text screening

Abu-Hammad O, Alduraidi H, Abu-Hammad S, Alnazzawi A, Babkair H, Abu-Hammad A et al. Side Effects Reported by Jordanian Healthcare Workers Who Received COVID-19 Vaccines. Vaccines. 2021;9(6):577. *Excluded based on outcome (adverse events).*

Abu Jabal K, Ben-Amram H, Beiruti K, Batheesh Y, Sussan C, Zarka S et al. Impact of age, ethnicity, sex and prior infection status on immunogenicity following a single dose of the BNT162b2 mRNA COVID-19 vaccine: real-world evidence from healthcare workers, Israel, December 2020 to January 2021. Eurosurveillance. 2021;26(6). *Excluded based on outcome (immunogenicity).*

Blumenthal K, Robinson L, Camargo C, Shenoy E, Banerji A, Landman A et al. Acute Allergic Reactions to mRNA COVID-19 Vaccines. JAMA. 2021;325(15):1562. *Excluded based on outcome (adverse events).*

Boyarsky B, Werbel W, Avery R, Tobian A, Massie A, Segev D et al. Immunogenicity of a Single Dose of SARS-CoV-2 Messenger RNA Vaccine in Solid Organ Transplant Recipients. JAMA. 2021;325(17):1784. *Excluded based on outcome (immunogenicity).*

Callegaro A, Borleri D, Farina C, Napolitano G, Valenti D, Rizzi M et al. Antibody response to SARS‐ CoV‐ 2 vaccination is extremely vivacious in subjects with previous SARS‐ CoV‐ 2 infection. Journal of Medical Virology. 2021;93(7):4612-4615. *Excluded based on outcome (immunogenicity).*

Chen G, Li X, Sun M, Zhou Y, Yin M, Zhao B et al. COVID-19 mRNA Vaccines Are Generally Safe in the Short Term: A Vaccine Vigilance Real-World Study Says. Frontiers in Immunology. 2021;12. *Excluded based on outcome (adverse events).*

Christie A, Henley S, Mattocks L, Fernando R, Lansky A, Ahmad F et al. Decreases in COVID-19 Cases, Emergency Department Visits, Hospital Admissions, and Deaths Among Older Adults Following the Introduction of COVID-19 Vaccine — United States, September 6, 2020–May 1, 2021. MMWR Morbidity and Mortality Weekly Report. 2021;70(23):858-864. *Excluded based on outcome (not effectiveness).*

Cirillo N. Reported orofacial adverse effects of COVID- 19 vaccines: The knowns and the unknowns. Journal of Oral Pathology & Medicine. 2021;50(4):424-427. *Excluded based on outcome (adverse events).*

Collier A, McMahan K, Yu J, Tostanoski L, Aguayo R, Ansel J et al. Immunogenicity of COVID-19 mRNA Vaccines in Pregnant and Lactating Women. JAMA. 2021;325(23):2370. *Excluded based on outcome (immunogenicity).*

Damiani G, Allocco F, Malagoli P. COVID- 19 vaccination and patients with psoriasis under biologics: real- life evidence on safety and effectiveness from Italian vaccinated healthcare workers. Clinical and Experimental Dermatology. 2021;46(6):1106-1108. *Excluded based on outcome (not effectiveness).*

Dean N. Hospital admissions due to COVID-19 in Scotland after one dose of vaccine. The Lancet. 2021;397(10285):1601-1603. *Excluded based on outcome (not effectiveness).*

Fertel B, Milk J, Simon E, Muir M, Smalley C. COVID-19 vaccine adverse reactions bring patients to emergency departments. The American Journal of Emergency Medicine. 2021;. *Excluded based on outcome (adverse events).*

Friedrich S, Friede T. Causal inference methods for small non-randomized studies: Methods and an application in COVID-19. Contemporary Clinical Trials. 2020;99:106213. *Excluded based on article type (not primary research).*

Gee J, Marquez P, Su J, Calvert G, Liu R, Myers T et al. First Month of COVID-19 Vaccine Safety Monitoring — United States, December 14, 2020–January 13, 2021. MMWR Morbidity and Mortality Weekly Report. 2021;70(8):283-288. *Excluded based on outcome (not effectiveness).*

Geers D, Shamier M, Bogers S, den Hartog G, Gommers L, Nieuwkoop N et al. SARS-CoV-2 variants of concern partially escape humoral but not T-cell responses in COVID-19 convalescent donors and vaccinees. Science Immunology. 2021;6(59):eabj1750. *Excluded based on outcome (immunogenicity).*

Hodgson S, Mansatta K, Mallett G, Harris V, Emary K, Pollard A. What defines an efficacious COVID-19 vaccine? A review of the challenges assessing the clinical efficacy of vaccines against SARS-CoV-2. The Lancet Infectious Diseases. 2021;21(2):e26-e35. *Excluded based on article type (not primary research).*

Lee E, Cines D, Gernsheimer T, Kessler C, Michel M, Tarantino M et al. Thrombocytopenia following Pfizer and Moderna SARS- CoV - 2 vaccination. American Journal of Hematology. 2021;96(5):534-537. *Excluded based on outcome (adverse events).*

Lee Y, Lim S, Lee J, Lim J, Kim M, Kwon S et al. Adverse Reactions of the Second Dose of the BNT162b2 mRNA COVID-19 Vaccine in Healthcare Workers in Korea. Journal of Korean Medical Science. 2021;36(21). *Excluded based on outcome (adverse events).*

Levine-Tiefenbrun M, Yelin I, Katz R, Herzel E, Golan Z, Schreiber L et al. Initial report of decreased SARS-CoV-2 viral load after inoculation with the BNT162b2 vaccine. Nature Medicine. 2021;27(5):790-792. *Excluded based on outcome (viral load).*

Lewnard J, Patel M, Jewell N, Verani J, Kobayashi M, Tenforde M et al. Theoretical Framework for Retrospective Studies of the Effectiveness of SARS-CoV-2 Vaccines. Epidemiology. 2021;32(4):508-517. *Excluded based on article type (not primary research).*

Lin T, Liao S, Lai C, Paci E, Chuang S. Effectiveness of non-pharmaceutical interventions and vaccine for containing the spread of COVID-19: Three illustrations before and after vaccination periods. Journal of the Formosan Medical Association. 2021;120:S46-S56. *Excluded based on article type (modelling study).*

Lu J, Wen X, Guo Q, Ji M, Zhang F, Wagner A et al. Sensitivity to COVID-19 Vaccine Effectiveness and Safety in Shanghai, China. Vaccines. 2021;9(5):472. *Excluded based on outcome (attitudes).*

Milman O, Yelin I, Aharony N, Katz R, Herzel E, Ben-Tov A et al. Community-level evidence for SARS-CoV-2 vaccine protection of unvaccinated individuals. Nature Medicine. 2021;. *Excluded based on outcome (not effectiveness).*

Monin L, Laing A, Muñoz-Ruiz M, McKenzie D, del Molino del Barrio I, Alaguthurai T et al. Safety and immunogenicity of one versus two doses of the COVID-19 vaccine BNT162b2 for patients with cancer: interim analysis of a prospective observational study. The Lancet Oncology. 2021;22(6):765-778. *Excluded based on outcome (adverse events and immunogenicity).*

Patel M, Bergeri I, Bresee J, Cowling B, Crowcroft N, Fahmy K et al. Evaluation of post-introduction COVID-19 vaccine effectiveness: Summary of interim guidance of the World Health Organization. Vaccine. 2021;39(30):4013-4024. *Excluded based on article type (not primary research).*

Salmerón Ríos S, Mas Romero M, Cortés Zamora E, Tabernero Sahuquillo M, Romero Rizos L, Sánchez‐ Jurado P et al. Immunogenicity of the BNT162b2 vaccine in frail or disabled nursing home residents: COVID‐ A study. Journal of the American Geriatrics Society. 2021;69(6):1441-1447. *Excluded based on outcome (immunogenicity).*

Song J, Cheong H, Kim S, Lee S, Kim S, Noh J et al. Early Safety Monitoring of COVID-19 Vaccines in Healthcare Workers. Journal of Korean Medical Science. 2021;36(15). *Excluded based on outcome (adverse events).*

Sørvoll I, Horvei K, Ernstsen S, Lægreid I, Lund S, Grønli R et al. An observational study to identify the prevalence of thrombocytopenia and anti‐ PF4/polyanion antibodies in Norwegian health care workers after COVID‐ 19 vaccination. Journal of Thrombosis and Haemostasis. 2021;19(7):1813-1818. *Excluded based on outcome (adverse events).*

Tougeron D, Hentzien M, Seitz-Polski B, Bani-Sadr F, Bourhis J, Ducreux M et al. Severe acute respiratory syndrome coronavirus 2 vaccination for patients with solid cancer: Review and point of view of a French oncology intergroup (GCO, TNCD, UNICANCER). European Journal of Cancer. 2021;150:232-239. *Excluded based on article type (not primary research).*

Vilches T, Zhang K, Van Exan R, Langley J, Moghadas S. Projecting the impact of a two-dose COVID-19 vaccination campaign in Ontario, Canada. Vaccine. 2021;39(17):2360-2365. *Excluded based on article type (modelling study).*

Zhao H, Souders C, Carmel M, Anger J. Low Rates of Urologic Side Effects Following Coronavirus Disease Vaccination: An Analysis of the Food and Drug Administration Vaccine Adverse Event Reporting System. Urology. 2021;153:11-13. *Excluded based on outcome (adverse events).*

Supplementary Table. Vaccine effectiveness by outcome among included studies.

Vaccine Effectiveness	N	Mean	Min	Max	Mean of 95% CI Standard Deviation
**Fully vaccinated (2 doses)**					
**Against infection**	17	78.58 %	61.00 %	95.00 %	61.53 % - 86.47 %
**Against hospitalization**	7	88.67 %	72.00 %	97.20 %	71.46 % - 93.60 %
**Against death**	3	92.37 %	86.00 %	96.70 %	75.20 % - 94.90 %
**Total**	**27**	**82.73 %**	**61.00 %**	**97.20 %**	**65.62 % - 89.26 %**
**Partially vaccinated (1 dose)**					
**Against infection**	12	61.25 %	16.00 %	84.00 %	43.25 % - 72.33 %
**Against hospitalization**	4	70.00 %	37.00 %	91.00 %	56.00 % - 77.50 %
**Against death**	3	50.67 %	46.00 %	55.00 %	36.33 % - 59.00 %
**Total**	**19**	**61.42 %**	**16.00 %**	**91.00 %**	**44.84 % - 71.32 %**
**Total (both fully and partially vaccinated)**	**46**	**73.93 %**	**16.00 %**	**97.20 %**	**57.04 % - 81.85 %**

Supplementary Figure. Quality assessment of included studies by study design.

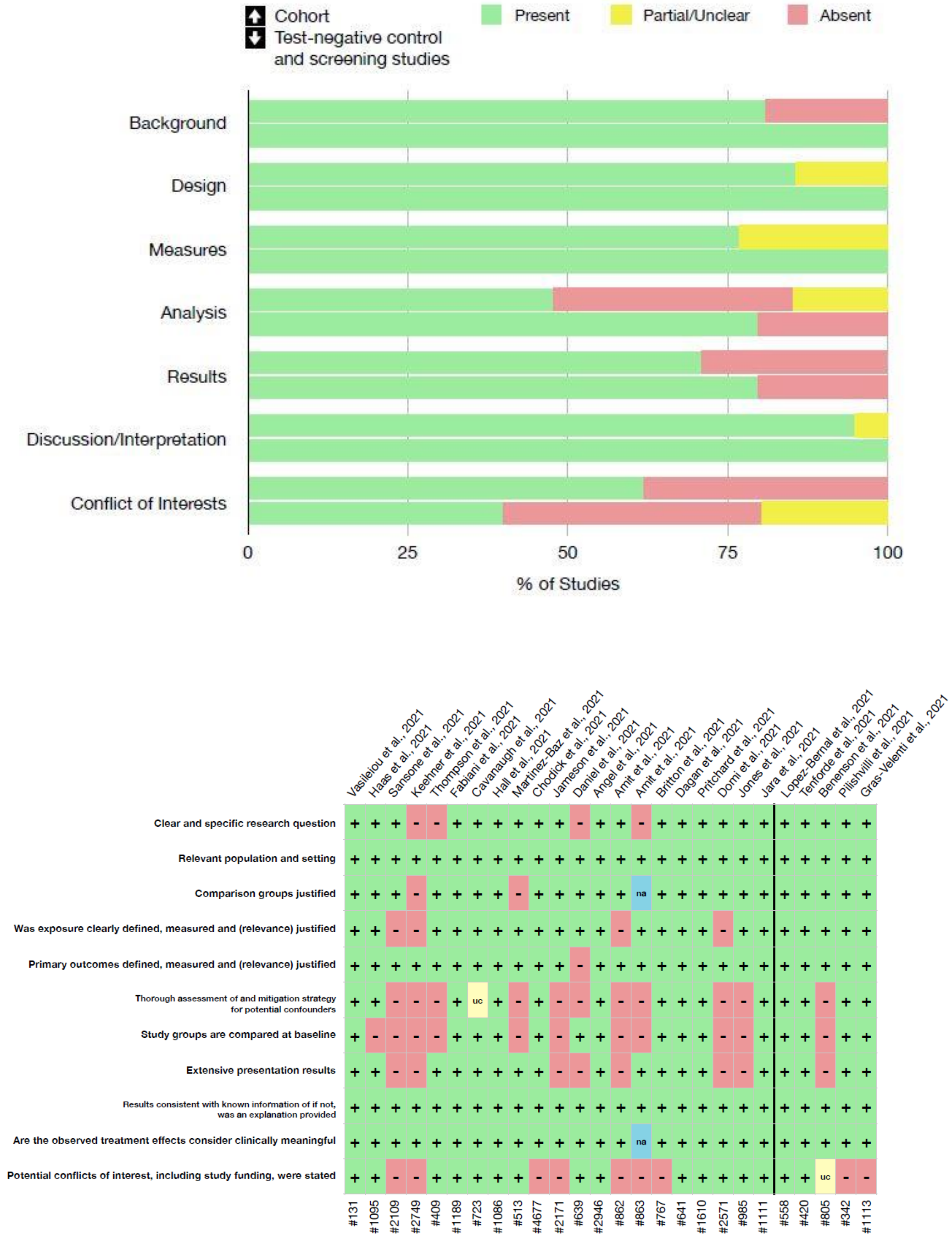

PRISMA checklist

Click here to access/download

Supporting Information

PRISMA_2020_checklist (1).docx
