# Supplementary material for: A systematic review of methodological approaches for evaluating real-world effectiveness of COVID-19 vaccines: Advising resource-constrained settings
Source: PLoS One. 2022 Jan 11;17(1):e0261930. doi: 10.1371/journal.pone.0261930 (PMC8752025; doi:10.1371/journal.pone.0261930)
Supplement: Attachment — Submitted filename: Response to reviewers - 25Nov2021.docx [file pone.0261930.s005.docx]

**Response to reviewers**

A systematic review of methodological approaches for evaluating real-world effectiveness of COVID-19 vaccines: advising resource-constrained settings

*Reviewer 1*

In this rapid review, Teerawattananon and colleagues summarize methodological approaches for evaluating real-world effectiveness of COVID-19 vaccines, with a particular focus on the need for these types of studies in low- and middle-income countries (LMICs).

As a general comment, the data from this review are under-analyzed. Most presented results are unrelated to stated research objective (to summarise methodological approaches being used to study real-world COVID-19 vaccine effectiveness). As the focus of this review is on methodological approaches, further details on study methodology should be summarized in the tables and text.

Figure 3, in particular, is misleading and not a stated objective of this rapid review. VE estimates will vary depending on study design, population, time period, etc. A critical assessment of the methodological biases present in these studies has not been provided on a study-by-study basis, which would allow for appropriate interpretation of these VE estimates. The focus of Results and Discussion should instead be on Figure 4. Additional methodological details should be added to Tables 1 and 2. The Introduction should focus more on methodological issues in conducting real-world VE studies, rather than a general discussion of COVID-19 vaccination strategies.

Finally, it is unclear what this systematic review adds to the body of literature. As mentioned in this review, the WHO recently published interim guidance on conducting VE studies in LMIC, which recommends the test-negative design as the most efficient and feasible method for LMICs. It also already summarizes key features of COVID-19 VE studies in its ‘Landscape of observational study designs on the effectiveness of COVID-19 vaccination’ evergreen document.

***Response:*** We would like to thank the reviewer for providing helpful direction on the focus of this review. We have revised the introduction, results and discussion section to discuss methodological issues in further detail, and we have limited discussion of the vaccine effectiveness results, given the extensive literature on this topic, as highlighted by both reviewers.

We believe that there is still merit in conducting this review to complement the WHO interim guidance and landscape of studies, as it illustrates the methodological designs used in peer-reviewed literature, as well as highlighting key gaps and weaknesses in current study design. This current review is the first of its kind to focus on the methodological approaches used in vaccine effectiveness studies. It will complement the WHO guidance and the evergreen document, as this review can provide concrete evidence on what has been done in real-world studies.

Specific comments:
1. Please update search strategy. Several recently published COVID-19 VE studies are missing from your review. The WHO landscape of observational studies document includes 90+ published studies (140+ when preprints included) where the main study outcome is ‘effectiveness’ but your review only includes 26 studies. Examples of missing citations (not a complete list):
a. Andrejko et al. Prevention of COVID-19 by mRNA-based vaccines within the general population of California. Clin Infect Dis. 2021 Jul 20. <https://doi.org/10.1093/cid/ciab640>
b. Chung et al. Effectiveness of BNT162b2 and mRNA-1273 covid-19 vaccines against symptomatic SARS-CoV-2 infection and severe covid-19 outcomes in Ontario, Canada: test negative design study. BMJ. 2021 Aug 10. <https://doi.org/10.1136/bmj.n1943>.
c. Pawlowski et al. FDA-authorized COVID-19 vaccines are effective per real-world evidence synthesized across a multi-state health system. Med (N Y). 2021 Jun 29. <https://doi.org/10.1016/j.medj.2021.06.007>.
d. Monge et al. Direct and Indirect Effectiveness of mRNA Vaccination against Severe Acute Respiratory Syndrome Coronavirus 2 in Long-Term Care Facilities, Spain. Emerg Infect Dis. 2021 Jul 27. <https://doi.org/10.3201/eid2710.211184>.
e. Shrotri et al. Vaccine effectiveness of the first dose of ChAdOx1 nCoV-19 and BNT162b2 against SARS-CoV-2 infection in residents of long-term care facilities in England (VIVALDI): a prospective cohort study. Lancet Infect Dis. 2021 Jun 23. <https://doi.org/10.1016/S1473-3099(21)00289-9>.

***Response:*** Thank you to both reviewers for proposing the additional papers for inclusion. There are a few reasons to explain the missing papers. First, our original search was run until 16 June, with additional papers added on an ad-hoc basis until 07 July. Second, we searched relevant publications from MEDLINE (via PubMed) and Scopus databases, and there is time lag between papers published in individual journals and their availability in these databases. This discrepancy would not be significant for systematic reviews of other subjects, but given the nature of the COVID-19 pandemic, there were several vaccine effectiveness studies on COVID-19 vaccine published in a week.

As such, we revised the search dates to inception until 07 July and carefully reviewed all newly identified abstracts. This process identified 10 of the papers proposed by the reviewers, 7 of which met our inclusion criteria and were included in our revised analysis. 3 papers did not meet our inclusion criteria and were excluded. Of the remaining 18 papers noted by the reviewers, 12 were indexed in PubMed/Scopus after the cut-off date of 07 July for our search. An additional 6 papers met our inclusion criteria and were indexed in PubMed before 07 July, but not identified in the search. We suspect that this is due to only using the MeSH term “COVID-19 Vaccines” and not including free text terms for each of the COVID-19 vaccines. We included these 6 additional papers in the analysis and amended our methods section to note that additional papers were identified through consultation with experts in the field.

Please see the summary in the table below for the list of papers specified by the reviewers and reason for inclusion/exclusion in our revised analysis.

| 1) Indexed in databases after search cut-off date (NOT INCLUDED, 12) | 2) Identified in revised search (INCLUDED, 7) | 3) Missed in search, added into analysis (INCLUDED, 6) | 4) Excluded during full-text screening (NOT INCLUDED, 3) |
| --- | --- | --- | --- |
| Andrejko et al | Abu-Raddad et al (NEJM) | Chodick et al | Mateo-Urdiales et al (not effectiveness analysis by our definition: comparison before and after vaccination) |
| Abu-Raddad et al (Journal of Travel Medicine) | Azamgarhi et al | Garvey et al | Victor et al (insufficient detail on methods for inclusion) |
| Bianchi et al | Flacco et al | Gupta et al | Sheikh et al (sub-group analysis of an included study) |
| Chung et al | Harris et al | Jaiswal et al |  |
| Hyams et al | Knobel et al | Walsh et al |  |
| Khavi et al | Mazagatos et al | Zacay et al |  |
| Malinis et al | Tang et al |  |  |
| Menni et al |  |  |  |
| Monge et al |  |  |  |
| Pawlowski et al |  |  |  |
| Shrotri et al |  |  |  |
| Swift et al |  |  |  |

2. Methods. Were preprints included? Given the rapidly changing COVID-19 vaccine landscape, inclusion of only published, peer-reviewed articles in your rapid review may introduce a publication bias. This may explain in part the study characteristics presented in Table 2, with more VE studies published in certain HICs (e.g. Israel, UK, US), population groups that were prioritized for early vaccination (e.g. nursing home residents, healthcare workers) or earlier variants (e.g. Alpha).

***Response:*** As specified in the search strategy, we did not include pre-prints. Since the objective of this review is to analyse the methods of vaccine effectiveness studies, we have only included peer-review articles as a quality standard, especially given that most pre-prints are not published. As the reviewer correctly notes, synthesis of vaccine effectiveness estimates is not the primary focus of our study and we recognise that we are likely to encounter publication bias when reporting on vaccine effectiveness estimates if we only include pre-prints. We have therefore removed extensive discussion of vaccine effectiveness estimates from the paper.

We are aware that peer-reviewed studies do not accurately represent all VE studies conducted around the world. However, in the South-East Asia region, we observe many unpublished VE studies that are neither good enough for publication nor in line with the methodological recommendations by the WHO guidance. For example, we have seen instances of case-controlled studies without a real control group, instead using proportion of unvaccinated population and rate of infection in the community. These studies could bring serious bias to policy decision making. We therefore believe that it is important for this systematic review to only evaluate peer-reviewed publications, demonstrating the strong and weak points of methodologically rigorous studies, in order to encourage researchers to consider and use rigorous methods in future vaccine effectiveness studies.

3. Methods, page 4 “If effectiveness data were unclear, the study was not included in the comparison of effectiveness but was kept for the qualitative analysis of study design and methods.” Please clarify in what way the effectiveness data were unclear. Would these studies not be excluded based on your requirement for “peer-reviewed literature, as quality assurance for study design and reporting”?

***Response:*** Since we have removed discussion of vaccine effectiveness in the revisions of the paper, we have removed this sentence.

4. Methods, page 4, “For studies reporting incidence rate ratio (IRR), the formula (1-IRR)*100 was used to calculate vaccine effectiveness.” What about studies reporting other effect estimates (e.g. odds ratio)?

***Response:*** All studies either reported vaccine effectiveness or IRR.

5. Methods, page 4. The authors used the RELEVANT tool to asses study quality. While this may be a useful tool for reporting of observational studies, other tools (e.g. ROBINS-I: <https://methods.cochrane.org/bias/risk-bias-non-randomized-studies-interventions>) may be better suited to assess the risk of bias.

***Response:*** Thank you for bringing the ROBINS-I tool to our attention. After reviewing the ROBINS-I tool, we noted that this tool is commonly recommended for assessing the risk of bias in non-randomized studies of interventions, not for observational studies. The main concern for both non-randomized and observational studies is confounding bias that is mainly focused in both ROBINS-I and RELEVANT tools. However, ROBINS-I also focus on the concern of co-interventions, which we felt may be less relevant for assessing risk of bias of observational studies. In addition, given that the focus of our study is the methodology of the real world studies, as opposed to vaccine efficacy itself, we felt that the RELEVANT tool is more suitable for our study than the ROBINS-I tool, given that the former is primarily developed for assessing the methodological quality of real-life comparative effectiveness observational research.

6. Methods, page 4, “Qualitative analysis identified areas of limited evidence and highlighted opportunities to strengthen COVID-19 vaccine effectiveness study methodology.” Please provide further details as to what type of qualitative analysis was performed.

***Response:*** Text updated to “Qualitative analysis of results from using the RELEVANT tool identified areas of limited evidence and highlighted opportunities to strengthen COVID-19 vaccine effectiveness study methodology.”

7. Results. Please reverse the order of Table 1 (study-specific results) and Table 2 (summary results).

***Response:*** Thank you for this comment. We have revised the order.

8. Results. Please add the following methodological details to Tables 1 and 2:
a. How was vaccination status was assessed (e.g. self-report, registry, etc.)?
b. Was outcome assessment restricted to certain time periods (e.g. ≥14 days post-vaccination)?
c. How was outcome misclassification error minimized (e.g. restricted to specimens collected within 7-10 days post symptom onset)?
d. Which methods were used to adjust for confounding? Which confounders were included?
e. How were missing data handled?
f. Were analyses restricted to symptomatic cases?
g. Did the author’s restrict or adjust for prior infection?
h. How were other sources of biases minimized (e.g. healthcare seeking behaviour)?

***Response:*** Thank you for proposing additional items for our analysis. We have included the points listed above into table 1 (now table 2) and provided greater discussion of these points in our results.

9. Discussion, page 7, “Most studies did not calculate (or report) the sample size a priori.” Sample size considerations will depend on study design. This will be important for studies proposing prospective, primary data collection but may be less relevant for retrospective cohort studies relying on linked administrative data (millions of records), for which accuracy will be more important than precision.

***Response:*** Well noted and agreed. We have added text to reflect this point: “Second, most studies did not calculate (or report) the sample size a priori. Whilst this may be less relevant for retrospective cohort studies based on national databases, which often utilise thousands or millions of records, it is an important consideration for prospective study designs or smaller scale retrospective cohort studies. Since many LMICs are unlikely to be able to replicate the large-scale studies from HICs, calculating minimum sample size will be very important, and should account for differences in access to healthcare services and health seeking behaviour in LMICs, as compared with HICs.”.

10. Appendix. Search terms appear incomplete. MEDLINE search terms are missing “vaccine”, “vaccination”, “immunization” and “COVID-19” or “coronavirus” or “SARS-CoV-2”. Did you include study designs besides cohort, e.g. “test negative design”, “case control design”, “screening method”? Did you include search terms for different product types, e.g. “mRNA” or “viral vector” or “BNT162b2” or “mRNA-1273”?

***Response:*** We did not include search terms by product type and we suspect that this is the reason for missing 6 of the studies proposed by reviewers. We have noted this as a limitation in the discussion: “Consultation with experts identified six additional papers that were not captured by our search terms, and there may be other studies which we missed. However, because the focus of our review is methodology of studies and not an estimate of vaccine effectiveness, we believe that this is acceptable.”.

*Reviewer 2*

This article has an interesting premise, but they do not prove their thesis of what would work in LMICs. They state at the end that TND is the best for LMIC but that is also what WHO has stated in their guidance and they provide pros/cons such as sample size and cost for getting to that conclusion.

***Response:*** Thank you for your comment. We believe that it is important to state whether the results of the review do or do not support the conclusion from WHO. Our review provides further evidence to strengthen the WHO position.

1) many articles published in peer review prior to their cut off date are missing. these need to be included. I am not sure why the systematic review did not find these but they were highly publicized findings in the media for some of them.

***Response:*** Thank you for listing the missing papers – we have reviewed all papers against our inclusion criteria and updated our search. Please refer to the response to comment 2 from reviewer 1.

2) I have made comments throughout for your review.

- “However, to our knowledge, there is no systematic review of published real-world effectiveness study designs for COVID-19 vaccination” - there are multiple ones

***Response:*** Thank you for bringing these papers to our attention. We have updated the text and reference the papers proposed: “Whilst many studies have synthesised COVID-19 vaccine effectiveness estimates from observational studies, to our knowledge, there is no systematic review of published real-world effectiveness study designs for COVID-19 vaccination, to support LMICs to understand which study designs are most feasible to implement in their settings, and the advantages and drawbacks of different approaches.”.

- “We therefore included any type of observational study, including cohort studies (prospective and retrospective), case control studies, test-negative design case-control studies, and screening studies, but excluded randomised control trials (RCTs) and modelling studies” - what about regression discontinuity design?

***Response:*** We excluded regression discontinuity design as it is currently recommended for vaccine effectiveness studies in diseases with low incidence or for which there is a long time lag until the outcome (https://academic.oup.com/aje/article/188/6/987/5341176). We have added text to this effect in the methods.

- “Any disagreement was resolved by YT or TA.” - what does this mean? I assume it’s two authors but should spell out

***Response:*** Text updated: “Any disagreement was resolved by one of two reviewers (YT or TA).”.

- Data analysis - WHO has updated strobe for ve studies. it's in the guidance document you cite.

***Response:*** Thank you for pointing this out. We believe that we have reflected relevant considerations for COVID-19 studies, as noted in annex 4 of the WHO guidance document, in our data extraction form.

- Results - you are missing many studies (see reference section for list that i found quickly from our database

***Response:*** Thank you for providing us with these studies. Please see the response to comment 2 from reviewer 1.

- Figure 3 – you can’t combine ve for all vaccines together

***Response:*** Noted, thank you. Given the focus of our paper, we have removed this figure.

- “none of the test-negative design studies measured chronic disease status or respiratory viral infection” - what does this mean?

***Response:*** Chronic disease status and respiratory infection are likely to increase likelihood of testing for COVID-19 (as noted in the WHO guidance document), so there could be bias in the results if there are differences between cases and controls. Text has been updated: “No study in our review measured adherence to NPIs and none of the test-negative design studies measured chronic disease status or respiratory viral infection, which could bias likelihood of individuals seeking COVID-19 tests.”.

- “importance of advocating for real-world effectiveness studies on all approved COVID-19 vaccines and across diverse LMIC settings” - agree. note that WHO is collecting data here on the landscape

***Response:*** Thank you for the comment.

- “only three pre-prints from HICs report on the delta variant and only four registered studies, all in HICs, will assess variants” - there are many more than what you cite here by your cut off date. i'm not sure what a registered study is since there is no clinicaltrials equivalent website for observational studies.

***Response:*** These data were taken from the WHO landscape document when reviewed in July. We have updated the text and added the access date to the reference list. Similarly, “registered study” was used to reflect the term in the WHO landscape document (which notes that filters are applied for observational studies).

- “In summary, our review highlights the importance of local vaccine effectiveness data, and suggests that test-negative case control studies with sample size calculated a priori may be most practical to implement in LMICs...” - you dont actually make this case as you dont talk about sample size, cost, why this is the case. its what we at who concluded but you havne't proven

***Response:*** Thank you for your comment. We have updated the text to reflect findings from our review:

“In summary, our review highlights the importance of local vaccine effectiveness data, and in providing further guidance on important confounders and methods for managing missing data. Most vaccine effectiveness studies to date have been conducted in HICs with access to suggests that test-negative case control studies with sample size calculated a priori may be most practical to implement in LMICs, especially since reliable and interlinked databases for COVID-19 vaccination, diagnosis and treatment. Such databases often do not exist in LMICs, meaning that countries will be employing prospective study designs, requiring a priori calculation of sample size and a clear plan to manage and report on confounders and missing data.”

- Amit et al – this isn’t a VE study

***Response:*** Thank you for the comment. We have excluded the study (wrong outcome).

- Figure 3 - remove gray background so that the lines can be better seen. need to say what hte horizontal vs veritcal line means.

***Response:*** Figure removed.

- Figure 3 - should point out that dont have enough time to actually calculate this and even hospitalization is flawed too

***Response:*** Figure removed to focus on methodological issues (and not vaccine effectiveness).

- Reference 13 - you shoudl put hte complete reference here and link

***Response:*** Noted and updated, thank you.

- Reference 16 - shoudl be listed as WHO and have a link

***Response:*** Noted and updated, thank you.

3) it would be improved if you did a risk of bias assessment on each one rather than just outlining what was reported. you say confounders are but don't describe which ones need to be paid attention to by researchers. WHO's guidance also lays out these confounders, but it's unclear which ones really significantly change the results. our own findings shows that time and location are extremely important along with age.

***Response:*** Thank you for this comment. The quality assessment for each study is summarised in figure 3. Unfortunately from the results of our systematic review, we are unable to advise on which confounders are most important. In our discussion we highlight the importance of providing guidance to LMIC researchers on the most important confounders: “We also propose the inclusion of practical guidance on identifying important confounders for a given setting and management of missing data.”

4) excluding pre-print articles doesn't really make sense. this is because the methods and the bulk of information rarely changes. you cite only 3 pre-prints by that point, but there were many many more by july.

***Response:*** Thank you for this comment – please refer to the response to comment 2 from reviewer 1.

5) you cannot lump all findings together for all vaccines as the vaccines are performing quite differently.

***Response:*** Given the focus of our paper is the methodology, and not the vaccine effectiveness results themselves, we have removed the presentation of results from our analysis.
